# Deep learning–based cognitive impairment brain imaging analysis: New methods, new technologies, and new paradigms

**DOI:** 10.4103/NRR.NRR-D-25-00332

**Published:** 2025-12-30

**Authors:** Qingqin Xu, Jianwei Lu, Zhongfu Zhang, Dongsheng Xu, Chengxiang Guo

**Affiliations:** 1 College of Rehabilitation Science, Shanghai University of Traditional Chinese Medicine, Shanghai, China; 2 Engineering Research Center for Traditional Chinese Medicine Intelligent Rehabilitation, Ministry of Education, Shanghai, China; 3 Information Technology Center, Guangxi University of Chinese Medicine, Nanning, Guangxi Zhuang Autonomous Region, China

**Keywords:** Alzheimer’s disease, cognitive dysfunction, deep learning, image classification, ischemic stroke, lesion segmentation, magnetic resonance imaging, neuroimaging, object detection, Parkinson’s disease

## Abstract

Cognitive impairment arising from ischemic stroke, Alzheimer’s disease, and Parkinson’s disease presents distinct structural and network-level alterations. Brain magnetic resonance imaging offers a non-invasive and high-resolution approach to assess these changes, while deep learning provides powerful tools for automated analysis. Given that accurate lesion delineation, precise localization of abnormal regions, and reliable disease classification are fundamental to clinical decision-making. This review aims to explore the application of deep learning techniques to brain magnetic resonance imaging analysis of cognitive impairments caused by these disorders, with a focus on three core tasks: lesion segmentation, object detection, and image classification. Recent widely accepted findings indicate that ischemic stroke studies have achieved state-of-the-art lesion segmentation performance, with optimized U-shaped convolutional network (U-Net) and hybrid convolutional neural network-transformer models reaching Dice scores up to 0.911 in delineating focal damage. Alzheimer’s disease research has advanced classification and staging accuracy by more than 10% compared with unimodal baselines through three-dimensional convolutional neural network, Transformers, and multimodal fusion, enabling more precise detection of diffuse cortical atrophy. Parkinson’s disease imaging, despite lacking overt structural lesions, has leveraged ResNet and Vision Transformer backbones to identify subtle and spatially distributed abnormalities, improving early-stage differentiation. Persistent challenges include the scarcity of large, high-quality annotated datasets, substantial inter-site variability, high annotation costs, and limited interpretability, hindering clinical integration. Addressing these barriers will require advances in federated learning to mitigate data scarcity while preserving privacy, domain adaptation techniques to reduce inter-site variability, automated annotation, and low-resource training strategies to lower labeling costs, and explainable artificial intelligence to improve interpretability, thereby ensuring model robustness, privacy, and transparency. This review highlights emerging methods, innovative technologies, and novel paradigms that are redefining brain imaging analysis in cognitive impairment. Mechanistically, deep learning improves cognitive impairment analysis by integrating hierarchical and multiscale spatial features, modeling long-range functional connectivity disruptions, and fusing structural with functional imaging to better represent network-level pathology. In conclusion, aligning network architectures with disease-specific imaging characteristics and task requirements can greatly enhance the accuracy, robustness, and generalizability of magnetic resonance imaging analyses for cognitive impairment. Future work should focus on multimodal fusion, structure-function coupling, cross-disease evaluations, and embedding artificial intelligence tools into clinical workflows to support early detection, individualized treatment planning, and large-scale clinical adoption.

**Facts**

• Deep learning has greatly improved brain magnetic resonance imaging segmentation, detection, and classification technologies in the fields of stroke, Alzheimer’s disease, and Parkinson’s disease.

• Core challenges include data scarcity, inter-institutional variability, high annotation costs, and insufficient interpretability.

• The combination of deep learning, multimodal fusion, and explainable artificial intelligence holds promise for advancing early diagnosis and clinical translation.

**Open questions**

• In the context of ischemic stroke, Alzheimer’s disease, and Parkinson’s disease, how can we design a unified deep learning framework that accommodates each disease’s unique imaging features while enabling early detection and comparative analysis of cognitive impairment across different diseases?

• How should we integrate federated learning, domain adaptation, automated annotation, and explainable artificial intelligence technologies in the future to construct a cognitive impairment magnetic resonance imaging analysis system that ensures privacy protection and clinical reliability?

• How can we develop an end-to-end deep learning model capable of performing multiple tasks while possessing cross-modal fusion capabilities to enhance the accuracy and clinical utility of cognitive impairment diagnosis?

## Introduction

According to the most recent Global Burden of Disease report, cognitive impairments pose a significant and growing global health challenge, affecting more than 350 million people worldwide and substantially leadint to disability and a decreased quality of life (Mtambo et al., 2025). These impairments are typically characterized by impairments in memory, attention, executive processing, and language and are frequently observed in patients with neurological disorders such as ischemic stroke (Feigin et al., 2021; Wang et al., 2021), Alzheimer’s disease (Soncu-Büyükişcan, 2025), and Parkinson’s disease (Gerner et al., 2025). From a neurobiological perspective, these conditions illustrate the continuum of neural injury, repair, and regeneration, ranging from acute focal tissue loss after stroke to chronic neurodegeneration in Alzheimer’s disease to selective subcortical damage in Parkinson’s disease (Su et al., 2025). Foundational studies on neuroplasticity and repair have established that structural damage triggers complex cascades of degeneration and compensatory remodeling, which can now be tracked *in vivo* via modern neuroimaging. Magnetic resonance imaging (MRI), with its noninvasive and high-resolution capabilities, has thus become central to mapping both injury patterns and trajectories of neural recovery.

Given their shared association with cognitive impairment yet distinct neuroimaging signatures, ischemic stroke, Alzheimer’s disease, and Parkinson’s disease provide an ideal comparative framework for examining how disease-specific pathophysiology influences deep learning-based brain MRI analysis. Stroke typically presents with focal lesions, Alzheimer’s disease is characterized by diffuse cortical atrophy, and Parkinson’s disease involves subcortical network disruptions. These differences affect preprocessing pipelines, feature extraction strategies, and choices of network architecture. Historically, most studies have adhered to a lesion-structure-function model (Ryan et al., 2021), which links discrete injuries to functional deficits. However, increasing evidence suggests that cognitive impairment often arises from distributed network perturbations involving the hippocampus, prefrontal cortex, and striatum across multiple conditions (Slotnick, 2024; Joyce et al., 2025; Reinhold et al., 2025). This has sparked an ongoing debate over whether focal or network-level abnormalities more accurately explain cognitive outcomes, with conflicting findings reported across different cohorts. In summary, multicenter MRI studies reveal significant variability in acquisition protocols, image quality, and population characteristics, leading to contradictory results regarding model generalizability.

Moreover, advances in MRI acquisition have generated massive, multidimensional datasets that challenge traditional visual inspection and handcrafted feature engineering (Kaplan et al., 2023; Rundo and Militello, 2024). Although conventional machine learning has achieved moderate success, its reliance on manual feature selection limits scalability and robustness. These limitations highlight the necessity for automated, data-driven methods such as deep learning, which excel at hierarchical feature extraction, nonlinear pattern recognition, and cross-modal adaptation (Yakkundi et al., 2024). Since the seminal introduction of deep neural networks and optimization strategies by Hinton and Salakhutdinov (2011), deep learning has gained traction in biomedical imaging and has been increasingly applied to map cognitive impairment-related brain changes (García-Gutiérrez et al., 2024). Nevertheless, current research remains fragmented: many studies focus on single diseases, prioritize either segmentation or classification without integrating tasks, or overlook the translation of algorithms into clinical decision-making.

This review addresses these gaps by providing a task-oriented and disease-specific synthesis of deep learning applications in brain MRI for cognitive impairment. Unlike prior reviews, our contributions are threefold: (1) we systematically integrate segmentation, detection, and classification perspectives with disease-specific analyses across ischemic stroke, Alzheimer’s disease, and Parkinson’s disease; (2) we critically evaluate and compare model architectures, tracing the progression from convolutional neural networks (CNNs) and U-Nets to transformer-based and hybrid designs; and (3) we explicitly connect imaging-based deep learning to the broader goals of neural injury characterization, repair monitoring, and regenerative intervention planning. By bridging technical innovation with clinical neuroscience, this review aims to serve both as a methodological reference and as a roadmap toward integrating artificial intelligence (AI)-powered MRI analysis into neurorehabilitation and regenerative medicine.

## Search Strategy

**Figure 1:**
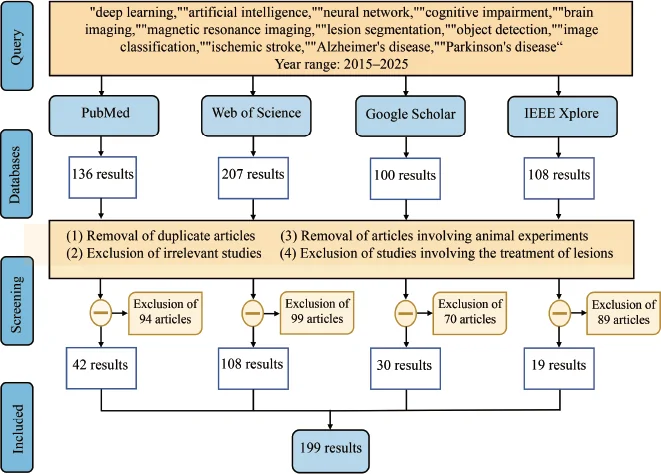
Flowchart of the systematic literature search and screening process across four databases (2015–2025). Studies involving animal experiments or treatment interventions were excluded to ensure consistency with the review’s focus on baseline human MRI data for deep learning-based neuroimaging analysis. Yellow boxes represent query keywords and screening steps; blue boxes represent databases; white boxes represent the number of included/excluded articles; arrows indicate the sequential flow of the screening process.

A comprehensive database search was conducted to identify peer-reviewed articles published between 2015 and 2025, utilizing search terms such as “deep learning,” “artificial intelligence,” “neural networks,” “cognitive impairment,” “brain imaging,” “magnetic resonance imaging,” “lesion segmentation,” “object detection,” “image classification,” “ischemic stroke,” “Alzheimer’s disease,” and “Parkinson’s disease.” The literature search was performed across multiple platforms, including PubMed, Web of Science, Google Scholar, and IEEE Xplore. The inclusion and exclusion criteria are summarized in **Additional Table 1**. The search yielded a total of 514 records (PubMed: 136; Web of Science: 207; Google Scholar: 100; IEEE Xplore: 108). After removing duplicates, irrelevant studies, animal experiments, and treatment-focused articles, we retrieved and included 199 studies in the qualitative analysis. A detailed flowchart outlining the search process is presented in **Figure 1**.

**Additional Table 1:** Inclusion and exclusion criteria

Inclusion criteria	Exclusion criteria
Deep learning techniques	Removal of duplicate articles
Cognitive impairments (ischemic stroke, Alzheimer's disease, Parkinson's disease)	Exclusion of irrelevant studies (e.g., traditional machine learning methods)
Magnetic resonance imaging (including variants)	Removal of articles involving animal experiments
Lesion segmentation, object detection, and classification	Exclusion of studies involving the treatment of lesions

## Deep Learning Foundation in Neuroimaging

### Fundamental architecture of deep learning

Deep learning is a term used to describe a class of machine learning approaches that use multilayer neural network architectures, also known as deep neural networks (Thapa et al., 2025). Deep neural networks have shown remarkable performance in representing complex high-dimensional data by learning multiple layers of nonlinear hidden features. Compared with shallow networks, deep architectures learn deep representations of raw data, which are abstract and generally invariant to variations in raw data and thus can reduce the reliance on manually designed features (Zhang et al., 2025b). This section describes the basic framework of deep learning models and summarizes representative architectures that are widely used in medical image analysis.

**Figure 2:**
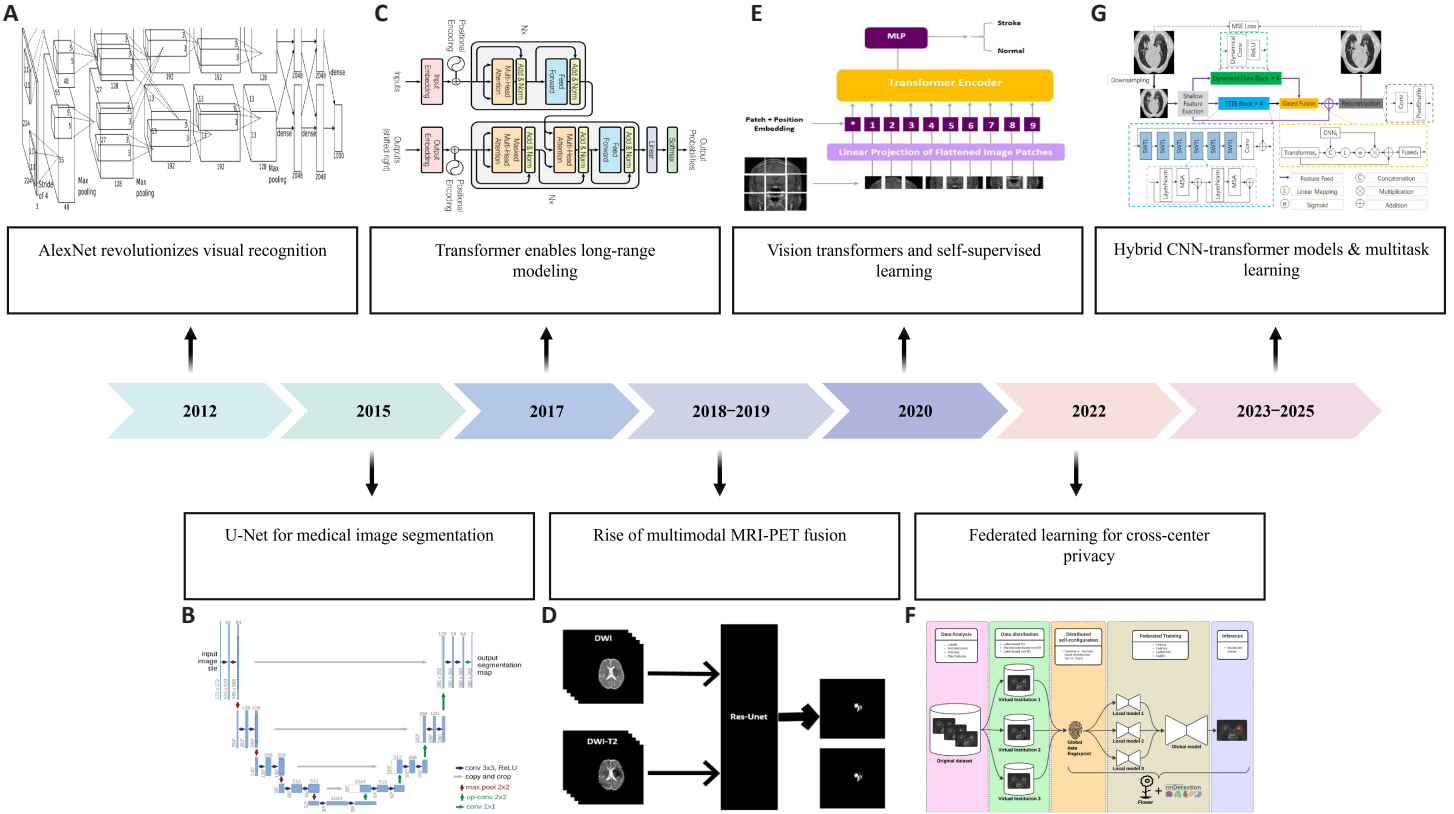
Timeline of key developments in deep learning for brain imaging analysis in cognitive impairment. (A) Introduction of AlexNet initiates a breakthrough in visual recognition (schematic summary adapted from Krizhevsky et al., 2012). (B) U-Net enables accurate medical image segmentation (Ronneberger et al., 2015). (C) Transformer introduces global attention for long-range modeling (schematic summary adapted from Vaswani et al., 2017). (D) Multimodal MRI-PET fusion methods emerge for Alzheimer’s disease diagnosis (Liu et al., 2019). (E) Vision Transformers and self-supervised learning improve feature abstraction with less annotation (Almufareh et al., 2023). (F) Federated learning is adopted for privacy-preserving, cross-center neuroimaging analysis (Rashidi et al., 2024). (G) Hybrid CNN-Transformer models and multitask learning support integrated structure recognition (Qin et al., 2025). Panels A and C are schematic summaries redrawn by the authors based on Krizhevsky et al. (2012) and Vaswani et al. (2017). Panels B, D, F, and G are reproduced with permission from Springer Nature (© 2015, © 2019, © 2024, and © 2025 Springer-Verlag London Ltd.). Panel E is adapted from an open-access article licensed under a Creative Commons Attribution License (CC BY). CNN: Convolutional neural network; MRI: magnetic resonance imaging; PET: positron emission tomography; U-Net: U-shaped convolutional network; ViT: vision transformer.

**Figure 3:**
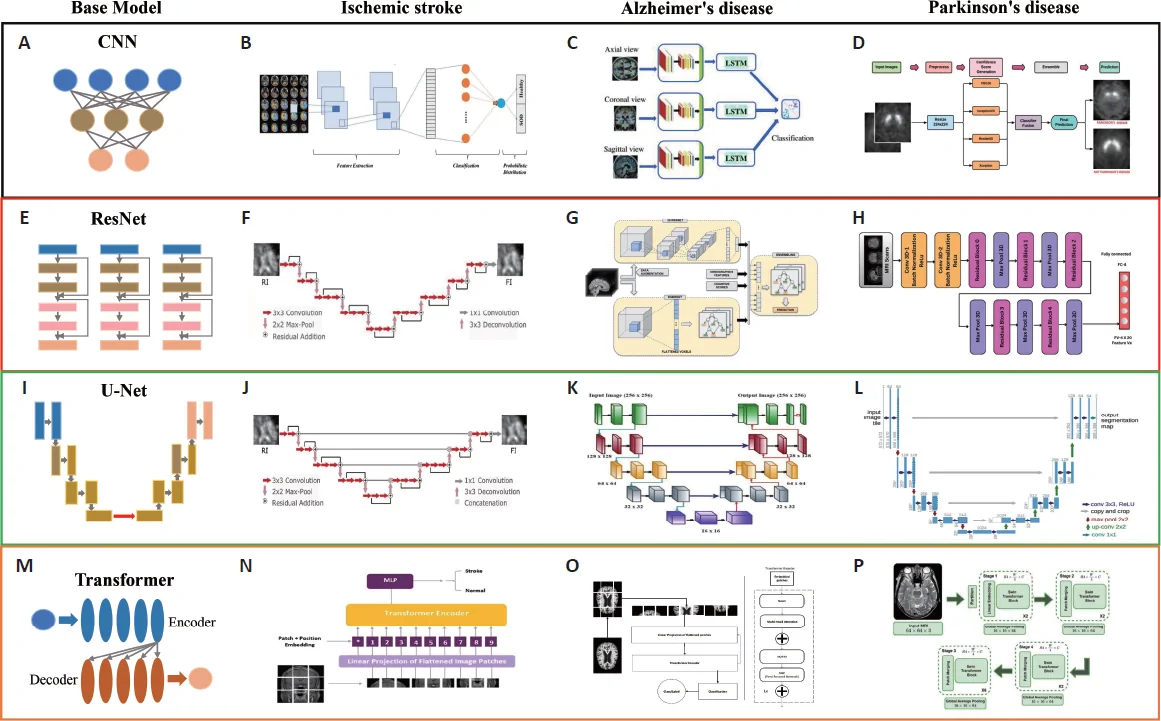
Representative deep learning architectures and their disease-specific applications. The first column (A, E, I, M) illustrates the four base models: CNN, ResNet, U-Net, and transformer. The subsequent columns present representative applications of each model to ischemic stroke (B, F, J, N) (Adlung et al., 2021; Dasari et al., 2023; Abbaoui et al., 2024), Alzheimer’s disease (C, G, K, O) (Hazarika et al., 2022; Nguyen et al., 2022; Almufareh et al., 2023; Hussain et al., 2025), and Parkinson’s disease (D, H, L, P) (Kurmi et al., 2022; Priyadharshini et al., 2024; Hussain et al., 2025), respectively. Yellow blocks indicate input layers, blue blocks represent convolutional feature extraction stages, purple blocks denote transformer encoder modules, green blocks represent output or classification stages, and arrows indicate data flow directions. Panels B, D, G, N, and O are adapted from open-access articles licensed under a Creative Commons Attribution License (CC BY). Panel C is reproduced with permission from SPIE (© 2021, Society of Photo-Optical Instrumentation Engineers). Panels F and J are reproduced with permission from Wiley (© 2021, John Wiley & Sons Ltd.). Panels H, K, L, and P are reproduced with permission from Springer Nature (© 2015, © 2022, © 2024, and © 2025 Springer-Verlag London Ltd.). CNNs: Convolutional neural networks; ResNets: residual networks; U-Net: U-shaped convolutional network.

To offer a more detailed historical perspective, **Additional Table 2** and **Figure 2** illustrate the chronology of significant events in the application of deep learning to brain imaging for cognitive impairment from 2015 to 2025, showing the corresponding paradigm transitions in network architecture, modality, and learning strategy. As shown in **Figure 3**, this review focuses on applications of deep learning in medical image processing. Specifically, CNNs are among the most widely used deep neural network architectures (Vakalopoulou et al., 2023). The basic architecture of CNNs consists of convolutional layers for extracting spatial features, activation functions for adding nonlinear layers, pooling layers for reducing dimensions and compressing features, and fully connected layers for classification tasks (Perumal et al., 2024). By utilizing local receptive fields and parameter-sharing strategies, CNNs greatly reduce model parameters and computational costs and are widely used in lesion segmentation, object detection, and image classification tasks. In recent years, researchers have proposed many advanced architectures to enable models to better learn long-range dependencies and extract multiscale features. For example, residual networks (ResNets) use residual connections to alleviate the vanishing gradient problem and facilitate deeper network training (Ni et al., 2025). U-shaped convolutional networks (U-Nets) use a symmetric encoder-decoder architecture combined with skip connections to preserve spatial information and enhance semantic representation, and have thus been widely used as mainstream models for medical image segmentation tasks (Pan et al., 2025). Furthermore, transformer architectures and their variants use a self-attention mechanism that can capture both local and global features simultaneously and therefore have shown good modeling performance when handling medical images with complex structures (Wu et al., 2025). In summary, deep learning models can effectively extract and transform complex features from medical images and provide a technical basis for fully automated brain MRI analysis in the context of cognitive impairment.

**Additional Table 2:** Comparison of emerging deep learning technologies for medical brain imaging analysis

Studies	Models	Key advantages	Challenges	Conclusions and clinical application
Ronneberger et al., 2015	U-Net	High segmentation accuracy	Limited global context modeling	The U-Net was primarily applied to ischemic stroke lesion segmentation, advancing precise medical image segmentation.
Vaswani et al., 2017	Transformer- based models	Captures long-range dependencies	Require large datasets	The transformer, based solely on attention, was first developed for NLP and later inspired imaging studies in cognitive impairment by enabling long-range dependency modeling.
Liu et al., 2019	Multimodal deep learning	Integrates structural and functional information	Require multimodal data availability	Multimodal deep learning was applied to automatically segment acute ischemic stroke lesions, integrating structural and functional imaging information.
Almufareh et al., 2023	Vision Transformer	Less need for handcrafted features	Need large-scale pretraining	Patch embedding with a Transformer encoder enables multi-class MRI classification in AD, reducing the reliance on handcrafted features.
Rashidi et al., 2024	Federated learning	Preserves data privacy	Communication overhead and model heterogeneity	Federated learning enables cross-center medical imaging analysis while preserving data privacy.
Qin et al., 2025	Hybrid models	Combine local and global features	Complexity in training and optimization	Hybrid models support lesion detection and classification, integrating both local and global feature learning.

This table summarizes representative emerging deep learning technologies in brain imaging, with relevance to cognitive impairment (2015-2025). AD: Alzheimer's disease; MRI: magnetic resonance imaging; NLP: natural language processing; U-Net: U-shaped convolutional network.

### How deep learning works

**Figure 4:**
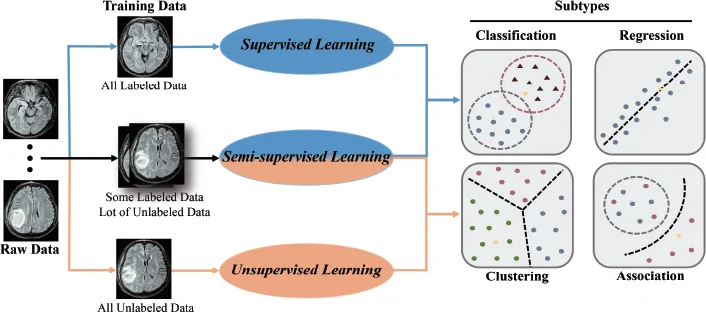
Learning paradigms in medical imaging: supervised, semi-supervised, and unsupervised learning. Blue arrows indicate the data flow from labeled data to supervised and semi-supervised learning tasks; orange arrows indicate the data flow from unlabeled data to semi-supervised and unsupervised learning tasks.

Deep learning is one of the most widely applied machine learning techniques. Its core principle involves constructing multilayer neural networks to extract data features, thereby performing complex tasks such as image segmentation, object detection, and image classification. Depending on the learning paradigm, deep learning algorithms can be categorized into supervised learning, unsupervised learning, and semisupervised learning (Gryshchuk et al., 2025; Pantanowitz et al., 2025). As shown in **Figure 4**, supervised learning is the most commonly used learning paradigm, wherein models are trained using a large set of labeled data to learn the mapping between inputs and outputs, enabling accurate predictions when new data are encountered (Feng and Zhang, 2025). Unsupervised learning focuses on uncovering the underlying structure, patterns, or distribution of data from unlabeled samples (Duan et al., 2025). Semisupervised learning combines the generalization capacity of unsupervised learning with the discriminative power of supervised learning (Sun et al., 2025).

### Core deep learning tasks

**Figure 5:**
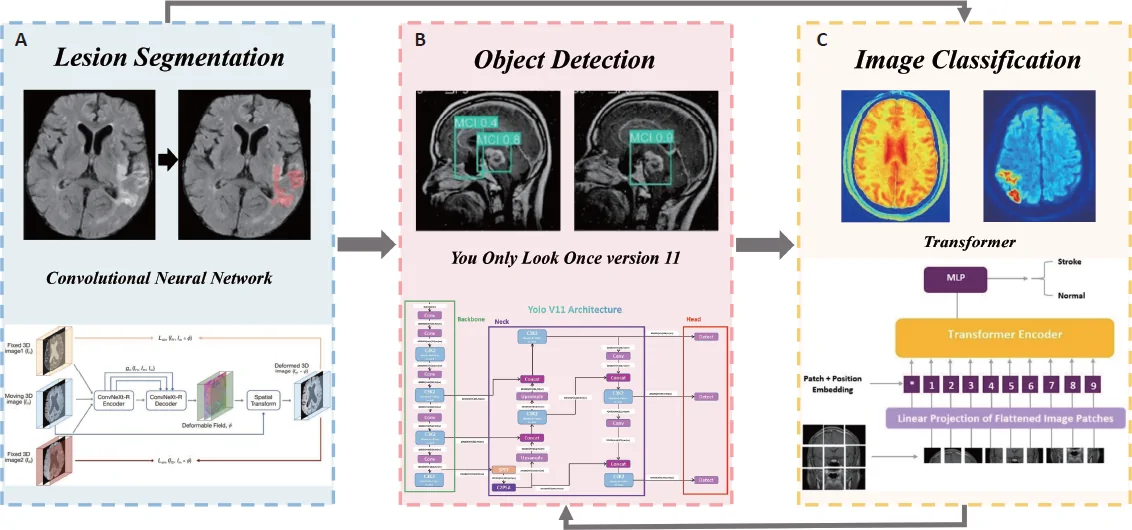
Deep learning frameworks for three major tasks in medical image analysis. (A) Lesion segmentation (Subbanna et al., 2019; Gui et al., 2024), (B) object detection (Hechkel and Helali, 2025), and (C) image classification (Abbaoui et al., 2024). The gray arrows indicate the potential for information flow and feedback between the tasks. Panels A–C are adapted from open-access articles licensed under a Creative Commons Attribution License (CC BY). In addition, one image in panel A is reproduced with permission from AME Publishing Company (© 2025, AME Publishing Company).

In the context of cognitive impairment research, deep learning has become a key technique in brain MRI analysis. Its applications are typically structured into three major task categories: lesion segmentation, object detection, and image classification. As shown in **Figure 5**, these tasks are both independent and interrelated within research workflows, individually focusing on precise lesion segmentation, automated localization of abnormal regions, and intelligent determination of disease states. The following sections provide a detailed overview of these three core tasks, outlining their objectives, representative models, and key applications in the context of cognitive impairment.

#### Lesion segmentation

Lesion segmentation is a fundamental task in medical image analysis that aims to accurately identify abnormal regions within brain MR images, such as infarcts, white matter hyperintensities, and atrophic areas. In the context of cognitive impairment, lesion segmentation provides crucial support for subsequent quantitative analysis and structural comparisons of brain regions. Deep learning models, particularly CNNs, U-Net, and various modified models, have demonstrated strong performance in automatically learning multiscale spatial features from complex neuroimaging data. In recent years, hybrid architectures that integrate the local modeling capabilities of CNNs with the global attention mechanisms of transformers have been proposed, significantly improving segmentation accuracy in cases involving indistinct lesion boundaries or small lesions. Lesion segmentation techniques have been successfully applied in tasks such as automatic segmentation of stroke lesions and hippocampal atrophy in Alzheimer’s disease (Bilello et al., 2015; Qin et al., 2025). Future research could focus on few-shot learning, multiscale feature fusion, and cross-modal joint segmentation strategies to increase robustness and adaptability to heterogeneous datasets.

#### Object detection

Object detection tasks aim to achieve spatial localization and categorical identification of regions of interest (ROIs) in medical images simultaneously, forming a crucial component of intelligent diagnostic systems. In cognitive impairment research, object detection is commonly used to rapidly localize structurally or functionally abnormal regions, such as the frontal lobe, hippocampus, and amygdala, which are core brain areas closely associated with cognitive functions. Current mainstream approaches include single-stage detection models, such as YOLO and SSD (Chen et al., 2022; Kang and Park, 2023), which provide high-speed inference and favorable real-time performance suitable for real-time assisted decision-making systems, and two-stage detection models, such as Faster R-CNN, which offer higher detection accuracy for recognizing complex brain structures, multiple lesions, or small lesions. Object detection technologies have demonstrated significant value in assisting clinicians with automatic annotation of ROIs, functional area comparisons, and surgical planning. However, this task still faces performance bottlenecks when dealing with complex lesions characterized by indistinct boundaries and substantial morphological variations. With advancements in federated learning and lightweight neural networks, object detection models are expected to achieve broader applicability in scenarios such as remote diagnostics, real-time analysis, and multicenter data processing (Zhong et al., 2023; Rashidi et al., 2024).

#### Image classification

Image classification refers to the task of applying deep learning models to classify the whole image or brain regions in an image into certain classes. Typically, image classification is applied in cognitive impairment tasks, including disease subtyping, severity grading, and disease progression prediction. Most image classification tasks use deep learning models, such as CNNs and ResNets, to extract global image features and then apply fully connected layers and softmax classifiers to the output classes (Papageorgiou et al., 2025). In recent years, many breakthroughs in attention mechanisms, multimodal information fusion, and contrastive learning approaches have greatly improved the accuracy, generalizability, and clinical applicability of image classification models (Xiong et al., 2025). Future work should extend these models to joint learning for multiple tasks and cross-modal information fusion to further improve the generalizability, interpretability, and clinical applicability of deep learning models (Khan et al., 2025a).

In summary, the three tasks jointly support an overall deep model for fully automated brain MRI segmentation for cognitive impairment. Segmentation tasks provide prior information for object detection and classification tasks, attracting attention to more brain ROI and classifying them more discriminatively. Conversely, classification tasks can provide feedback information to improve the attention mechanisms of object detection models, which form an enhancement loop among models.

## Deep Learning Methods for Brain Magnetic Resonance Imaging Analysis in Ischemic Stroke

### Lesion segmentation in ischemic stroke MR images via deep learning

Medical image segmentation is one of the key steps in the processing and analysis of medical images. Previous studies often used traditional image processing methods, such as thresholding, to perform pixelwise segmentation of lesions (Pham et al., 2000; Zhang et al., 2001). However, the segmentation accuracy of traditional methods is highly susceptible to factors such as image quality, threshold selection, and background complexity. This is particularly problematic when processing brain MR images with diverse shapes, uneven densities, variable positions, and ambiguous boundaries, where traditional methods often struggle to achieve precise segmentation (Kaesemann et al., 2014). In recent years, deep learning-based methods for medical image segmentation have advanced rapidly to increase segmentation accuracy and automation, demonstrating significant advantages in the segmentation of stroke-related MR images. This section explores classical CNNs and their variants, with a particular emphasis on the application of U-Net in medical image segmentation. Moreover, it delves into recent advancements in transformer-based models and hybrid models. Finally, the current research status, limitations, and future research directions concerning these models in stroke lesion segmentation are summarized.

#### Medical image segmentation methods based on classic convolutional neural network architectures

CNNs are foundational architectures in the field of image processing and are known for their ability to handle high-dimensional data efficiently, extract multiscale features, and autonomously learn spatial representations. These networks bypass the complexities of manual feature extraction and have been widely adopted for lesion segmentation in brain MR images. As shown in **Additional Table 3**, scholars explored the combination of different convolutional operations, using dilated convolutions to expand the receptive field and using transposed convolution to restore spatial resolution, further enhancing segmentation accuracy (Joshi and Gore, 2018; Yu et al., 2021). Winzeck et al. (2019) used an ensemble model composed of multiple CNN architectures trained on multiparametric MR images, demonstrating that this approach overcomes the limitations of a single CNN model and significantly improves the segmentation accuracy of acute infarct regions. These studies indicate that CNNs, through advanced network architectures, effectively enhance lesion segmentation performance, showing significant potential for application in brain image segmentation tasks.

**Additional Table 3:** Summary of CNN methods applied for lesion segmentation in ischemic stroke

Studies	Models	Innovative aspects	Research results	Practical significance
Joshi and Gore, 2018	CNNs, dilated convolutions	Semantic segmentation	DS: 0.85 JS: 0.78	Dilated and transposed CNNs enhance ischemic stroke lesion segmentation from MRI images.
Winzeck et al., 2019	Multiple CNNs	Ensemble CNN model	DS: 0.822 Precision: 0.832	CNN ensembles trained on multiparametric DWI enable accurate acute infarct segmentation with high agreement to manual outlines.
Xue et al., 2020	2.5D CNNs	Mixing attention mechanism and deep supervision	DSC: 0.745 Precision: 0.637 Recall: 0.582	A multimodal multi-path CNN system improves automated stroke lesion segmentation and approaches human-level accuracy.
Bal et al., 2023	3D CNNs	Connectivity among the different slices	DS (sub-acute): 0.87 DS (acute): 0.90	A two-pathway 3D CNN using multimodal MRI improves acute and sub-acute ischemic stroke lesion boundary segmentation.
Liu et al., 2019	15-layer 3D CNNs	Two symmetrical deep sub-networks	DS: 0.81 HD: 23.62 mm	A dual sub-network CNN with dense blocks and multi-kernel design achieves top performance in stroke MRI segmentation.
Karthik et al., 2019	FCNs	Leaky rectified linear unit	DS: 0.70	A deep supervised FCN with Leaky ReLU improves ischemic stroke lesion segmentation accuracy over existing methods.
Karthik et al., 2020a	FCNs, attention mechanism	Salient features of the lesion region	DS: 0.754	An attention-enhanced FCN improves ischemic lesion segmentation from multispectral MRI with higher accuracy.
Pérez Malla et al., 2019	CNNs, transfer learning, data augmentation	Lesion heterogeneity in location, shape, size, and intensity	17% improvement in DS over the baseline model	Data augmentation and post-processing improve CNN-based ischemic stroke lesion segmentation in MRI perfusion imaging.
Gómez et al., 2023	CNNs, cross-attention	Unbalanced tissue classes, variable shape, and texture	DS: 0.57 Precision: 0.67	A cross-attention autoencoder with deep supervision improves ischemic lesion delineation from multimodal MRI.
Geetha et al., 2025	CNNs, DeepLab-V3Plus	Geometric and moment features	MCC: 0.979	Automated stroke lesion segmentation enables correlation of imaging features with the modified Rankin Scale for outcome assessment.
Zhu and Sun, 2024	CNNs, feature attention module	Capturing long-term dependencies	DS: 0.613 IoU: 0.490	A 3D GFA-UNet with global feature attention improves stroke lesion segmentation by capturing long-term dependencies and context.
Hirsch et al., 2021	3D-D CNNs, TPMs, CRFs	Abnormal brain tissue	DS: 0.73	Deep volumetric networks with spatial priors improve brain MRI segmentation accuracy.
Ashtari et al., 2023	CNNs, NMF (Factorizers)	An end-to-end segmentation model	DS: 0.765 HD: 11.96 mm	Low-rank factorization improves efficiency and interpretability of stroke lesion segmentation.

This table summarizes representative CNN-based methods for ischemic stroke lesion segmentation, highlighting differences in imaging modalities, network architectures, and feature extraction strategies. These methods aim to improve segmentation accuracy, enable reliable quantitative lesion assessment, and ultimately enhance clinical decision-making and patient outcomes. CNNs: Convolutional neural networks; CRFs: conditional random fields; DS: dice score; DSC: dice similarity coefficient; FCNs: fully convolutional networks; HD: Hausdorff distance; loU: intersection over union; ISLES: ischemic stroke lesion segmentation challenge; JS: Jaccard score; MCC: Matthews correlation coefficient; MRI: magnetic resonance imaging; NMF: non-negative matrix factorization; TPMs: tissue probability maps; U-Net: U-shaped convolutional network; 3D-D CNNs: three-dimensional deep CNNs; 3D GFA-UNet: 3D global feature attention U-Net.

Complex medical MRI images encompass not only single-slice static images but also multi-slice and three-dimensional images, the latter of which are significantly more challenging to segment automatically. Xue et al. (2020) extended this work by developing multichannel 2.5D CNNs that combine multislice 2D planar information and local 3D contextual data. The proposed network achieved a better Dice coefficient than did naive Bayes, random forests, and traditional CNNs across three datasets and achieved improved segmentation accuracy and enhanced small lesion detection ability in cross-validation. To address the challenges of the accuracy of lesion segmentation and delineation of boundaries, Bal et al. (2023) used 3D CNNs with a dual-path design to integrate local and contextual information. The Dice similarity coefficient, sensitivity, and positive predictive value of the CNN models were compared on the ISLES 2015 dataset. Compared with existing CNN models, the proposed method achieves a higher Dice similarity coefficient, sensitivity, and positive predictive value.

To address the limitations of traditional CNNs in image segmentation tasks, including fixed input size constraints, spatial information loss, challenges in pixel-level prediction, and the inability to perform end-to-end training, Professor Jonathan Long introduced fully convolutional networks (FCNs) in 2015 (Pereira et al., 2019). FCNs replace the fully connected layers of CNNs with convolutional layers, allowing the network to receive an input of arbitrary size and produce an output of the same size. Since convolution and deconvolution can effectively preserve the structure of images, FCNs have been widely utilized to support end-to-end training. We refer to FCNs as innovative CNNs, as they represent an extension and enhancement of the application range of traditional CNNs (Abbasi et al., 2023). To reconstruct the ischemic lesion region, Karthik et al. (2019) presented a deep supervised FCN and achieved an average Dice coefficient of 0.70 on the ISLES 2015 dataset by adding the leaky rectified linear unit activation layer in the last two layers of the network. To further improve the accuracy of lesion region segmentation, Karthik et al. (2020a) added an attention mechanism to FCNs and attained an average Dice coefficient of 0.754 on the ISLES 2015 dataset, which was an increase of 7.6% compared with the state-of-the-art methods. Karthik et al. (2021) further improved the segmentation performance of FCNs on the ISLES 2015 dataset by refining the multilevel loss, feature integration, and attention mechanism, achieving an average Dice coefficient of 0.775.

To address the challenges faced by existing CNN architectures in segmenting ischemic stroke lesions, such as limited global context capture, insufficient local feature extraction, and low computational efficiency, researchers have gradually recognized that optimizing the network architecture or adjusting algorithms can offer superior performance compared to single CNN models. Pérez Malla et al. (2019) combined data augmentation, transfer learning, and postprocessing techniques with CNNs, achieving a 17% higher Dice score than the baseline model on the ISLES 2017 dataset. Gómez et al. (2023) proposed approaches such as a cross-attention mechanism, deep supervision, and weighted loss functions to improve the CNN-based autoencoder model, solve problems such as class imbalance and complex shapes of lesions well, and then realize the automatic localization and accurate segmentation of stroke lesions. Subsequently, Geetha’s team further improved the DeepLab-V3 model and proposed the DeepLab-V3Plus model. DeepLab-V3Plus enhances the multiscale feature processing ability and detail recovery ability well, and the experimental results show that its performance in the segmentation of acute ischemic stroke lesions is outstanding (Geetha et al., 2025). To enhance the accuracy, robustness, and consistency of the model, Zhu and Sun (2024) proposed a global feature attention module based on classical 3D CNNs, and then improved the segmentation precision by adding long-range dependencies and global context information. Hirsch et al. (2021) improved the accuracy of abnormal brain tissue segmentation, by adding three types of prior knowledge, including TPMs, CRFs and expanded receptive fields into 3D deep CNNs (3D-D CNNs). To increase the feature representation capability and learning efficiency of the model, Ashtari et al. (2023) proposed the factorizer model, and the experimental results on the ISLES 2022 dataset show that, compared with existing methods, the segmentation accuracy of the ISLES 2015 dataset has been significantly improved by adding nonnegative matrix factorization (NMF) and feature reuse techniques.

Overall, the image segmentation method based on a classical CNN architecture shows great potential in ischemic stroke lesion segmentation. However, many challenges still exist in current ISLES segmentation research, such as limited training data, high annotation costs, and a large amount of required computing resources (Luo et al., 2016). In the future, we could attempt to improve the performance of models and make them more applicable by introducing advanced devices, multimodal data integration, and self-supervised learning methods.

#### Medical image segmentation methods based on U-Net architectures

Although traditional CNNs have achieved certain breakthroughs in medical image segmentation applications, they are still limited by global context information, fine-grained segmentation of boundaries, and the range of the receptive field. To solve the above problems, Ronneberger et al. (2015) proposed the U-Net architecture at the MICCAI conference. U-Net uses a simple encoder-decoder architecture, and features extracted in the encoding process are combined with corresponding layers in the decoding process through skip connections, which can capture contextual information while maintaining spatial information. As shown in **Additional Table 4**, Yu et al. (2020) used the U-Net model to segment brain MR images, and the experimental results revealed that the segmentation performance of this model was better than that of existing clinical imaging segmentation methods. Lee et al. (2023) also conducted a stroke lesion segmentation experiment based on the U-Net architecture, and the experimental results revealed that the model can distinguish normal and lesion areas better than traditional segmentation models can.

**Additional Table 4:** Summary of U-Net methods applied for lesion segmentation in ischemic stroke

Studies	Models	Innovative aspects	Research results	Practical significance
Yu et al., 2020	U-Net	Using MRIs acquired at the initial presentation	DS: 0.58 Sensitivity: 0.74	Deep learning predicts subacute infarct lesions from baseline imaging, supporting treatment planning and clinical trial selection.
Lee et al., 2023	U-Net	Using brain structure as prior information	DS: 0.560 Precision: 0.604 Recall: 0.632 HD: 12.94 mm	A deep learning framework segments fine stroke-affected regions, enhancing brain modeling for non-invasive neuromodulation.
Winzeck et al., 2018	3D U-Net	Capturing 3^rd^ dimension information in MRI	DS: 0.55 Precision: 0.77	A 3D CNN-based U-Net improves ischemic stroke lesion segmentation with top performance in ISLES 2016/2017.
Joshi and Gore, 2018	U-Net, dilated and transposed convolutions	An encoder-decoder CNN with dilated convolution	DS: 0.85 JS: 0.78	Dilated and transposed CNNs enhance ischemic stroke lesion segmentation from MRI images.
Liu et al., 2019	U-Net, residual unit	Single-modality and multimodality images	DS: 0.891 HD: 1.51 mm	A deeper U-Net with residual modules improves information flow and segmentation accuracy.
Ibtehaz and Rahman, 2020	MultiResUNet	Introducing deeper convolutional layers	JS: 0.782	MultiResUNet enhances U-Net with improved robustness and accuracy, especially on challenging multimodal datasets.
Wong et al., 2022	U-Net, grouped convolutions	Incorporating rotation-reflection equivariance	DS: 0.85 Accuracy: 0.75	A rotation-reflection equivariant U-Net improves acute ischemic stroke segmentation and supports accurate outcome prediction.
Sheng et al., 2022	CADS-UNet	Mixing deep supervision and mixed loss	DSC: 0.556 Precision: 0.637 Recall: 0.582	CADS-UNet with cross-attention and deep supervision improves chronic stroke lesion segmentation on Tl-weighted MRI.
Hui et al., 2021	DPAC-UNet	Small lesions and blurred lesion boundaries	DSC: 0.592 Precision: 0.656 Recall: 0.599	DPAC-UNet enhances stroke lesion segmentation, particularly for small or blurred lesions.
Wang et al., 2023a	U-Net, attention mechanisms	A convolutional fusion encoding module	DSC: 0.619 Precision: 0.601	A convolution-attention fusion model improves segmentation, especially for small lesions with blurred boundaries.
Vupputuri et al., 2021	MCA-DN	Multi-path convolution	DSC: 0.773	MCA-DN with multi-path convolution attention improves stroke lesion segmentation and supports treatment planning.
Wong et al., 2021	U-Net, embedding network	Complementing the encoder-decoder module	DSC: 0.703 Precision: 0.806 Recall: 0.654	A spatial embedding network enhances encoder-decoder segmentation, improving stroke lesion accuracy on ATLAS.
Yu et al., 2023	SAN-Net	A self-adaptive normalization network	DS: 0.602 Recall: 0.657 F1-score: 0.625	SAN-Net with adaptive normalization and symmetry augmentation improves cross-site generalization for stroke lesion segmentation.

This table summarizes representative U-Net-based methods for ischemic stroke lesion segmentation, covering variations in network architecture, convolutional strategies, and feature enhancement techniques. These methods aim to improve segmentation accuracy, enable reliable lesion quantification, and support clinical diagnosis and treatment planning. CNN: Convolutional neural network; CRFs: conditional random fields; DS: dice score; DSC: dice similarity coefficient; F1: Fl-score; HD: Hausdorff distance; FCN: fully convolutional network; JS: Jaccard score; MCC: Matthews correlation coefficient; MRI: magnetic resonance imaging; nnU-Net: No-new-Net, self-adapting U-Net framework; TPMs: tissue probability maps; U-Net: U-shaped convolutional neural network.

To improve the performance of U-Net in handling medical images for different segmentation tasks, researchers have proposed different types of convolutions in U-Net, such as 3D convolutions, dilated convolutions, and grouped convolutions. Winzeck et al. (2018) proposed a 3D CNN-based U-Net architecture that captures contextual information from lesion regions to extract global features, successfully enhancing the automatic segmentation of ischemic stroke lesions. This approach achieved outstanding performance in the ISLES 2016 and 2017 challenges.

Joshi and Gore (2018) improved the accuracy of the model for complex lesion segmentation by adding dilated convolutions, achieving excellent performance on a test dataset with a Dice similarity coefficient of 0.85 and a Jaccard similarity coefficient of 0.78. Liu et al. (2019) proposed a deeper convolutional layer and residual module U-Net architecture to increase the segmentation accuracy of the model by improving the information flow and gradient propagation. To solve the problem of segmenting lesions of different sizes and shapes, Ibtehaz and Rahman et al. (2020) proposed MultiResUNet, which integrates multiresolution convolutions and residual connections. The proposed method improved the segmentation accuracy of the standard U-Net on five different datasets by 10.15%, 5.07%, 2.63%, 1.41%, and 0.62% (Ibtehaz and Rahman, 2020). Wong et al. (2022) integrated U-Net with grouped convolutions and achieved excellent performance in automatic segmentation of acute ischemic stroke lesions, and got a Dice coefficient of 0.85 on the test dataset.

To address the challenges faced by the existing U-Net architecture in segmenting ischemic stroke lesions, such as inadequate small lesion detection, limited local information capture, and poor generalizability, researchers have continuously refined or optimized the U-Net architecture to increase its performance. To enable the model to focus on critical lesion areas, Sheng et al. (2022) proposed the cross-attention and deep supervision U-Net (CADS-UNet) model, which integrates cross-spatial attention mechanisms, channel attention mechanisms, and deep supervision strategies to enhance the target detection capability of the model. To improve segmentation performance for small lesions and blurred boundaries, Hui et al. (2021) incorporated two independent paths into the U-Net architecture, proposing the dual-path attention compensation U-Net (DPAC-UNet), which achieved a 6% higher Dice score than the traditional single-path attention U-Net on the ATLAS dataset. Wang et al. (2023a) incorporated a channel attention mechanism into the U-Net framework, which demonstrated superior performance in handling small lesions and blurry boundaries, effectively improving segmentation accuracy and the ability to delineate edge details. Vupputuri et al. (2021) improved the U-Net architecture with an attention mechanism and proposed the multipath convolutional attention deep network (MCA-DN), which outperformed U-Net, attention U-Net, FCNs, TransUNet, and DeepLab in Dice score, sensitivity, specificity, and other metrics on the ISLES 2015 and ISLES 2017 datasets. Wong et al. (2021) used embedding networks into the U-Net architecture and used learnable downsamplers to improve feature representation, achieving an 11.7% Dice score improvement over the baseline architecture on the ATLAS dataset. To enhance the generalization ability of the model on different datasets, Yu et al. (2023) proposed a self-adaptive normalization network (SAN-Net), which adopted an adaptive normalization technique to effectively solve the problem of site-to-site variation in cross-site image segmentation, and used a symmetry-inspired data augmentation method to improve the generalization ability of the model.

In conclusion, U-Net-based methods are effective approaches for ischemic stroke MR image segmentation. However, the U-Net model still has the following limitations: weak small lesion detection ability, easy overfitting and poor generalization performance (Karthik et al., 2020b). Related studies can focus on these problems and attempt to solve them by applying methods such as multiscale feature fusion and data augmentation to improve the segmentation efficiency.

#### Medical image segmentation methods based on transformer architectures

Traditional CNNs and their variants (such as U-Net) have a small receptive field due to the convolution operation, and their capacity for modeling long-range dependencies is also limited, which leads to limitations in the field of small lesion segmentation and complex structure modeling. In 2017, the transformer model proposed by the Google research team broke through the limitations of the aforementioned issues with the self-attention method and global modeling approach. As shown in **Additional Table 5**, Karimi et al. (2022) used a transformer model, removing the convolution operation from two medical image segmentation datasets. The self-attention mechanism in the model improved the modeling of long-range dependencies. To further improve the segmentation performance of the model in the boundary region, Wu et al. (2023a) proposed TransRender, which introduces point-based rendering and a boundary feature-adaptive extraction mechanism. This method effectively solves the problem of boundary oversmoothing that often occurs in stroke lesion segmentation (Wu et al., 2023a).

**Additional Table 5:** Summary of pure transformer methods applied for lesion segmentation in ischemic stroke

Studies	Models	Innovative aspects	Research results	Practical significance
Karimi et al., 2022	Transformer	Self-attention between these patch embeddings	DSC: 0.878 HD: 0.871 mm	A transformer-based segmentation model outperforms FCNs and achieves high accuracy with limited labeled data.
Wu et al., 2023a	TransRender	Computing boundary features in a point-based rendering way	DSC (ATLAS): 0.598 Precision (ATLAS): 0.639 DSC (ISLES): 0.854 Precision (ISLES): 0.865	TransRender leverages transformers for boundary rendering to improve stroke lesion segmentation.
Ashtari et al., 2023	Transformer, NMF	Modeling long-range dependencies	DS: 0.765 HD: 11.96 mm	Factorizer with low-rank factorization improves stroke lesion segmentation with high accuracy and interpre tability.
Wu et al., 2024	Transformer, TAG	Employing a symmetric encoding-decoding structure	DSC (ATLAS): 0.585 HD (ATLAS): 39.56 mm DSC (ISLES): 0.839 HD (ISLES): 33.26 mm	FRPNet with twin attention and multi-dimensional pooling improves stroke lesion segmentation by capturing global-local features and details.
Jazzar et al., 2024	Transformer Dil-DenseUNet	DenseNet and dilated convolution	DSC (ISLES 2015): 0.80 ± 0.30 DSC (ISLES 2022): 0.81 ± 0.33	Transformer Dil-DenseUNet, combining DenseNet, dilated convolutions, and Transformers, achieves state-of-the-art stroke lesion segmentation.

This table summarizes representative pure Transformer-based methods for ischemic stroke lesion segmentation, focusing on their architectures, feature modeling strategies, and evaluation metrics. These methods leverage self-attention and long-range dependency modeling to improve segmentation accuracy, enhance lesion boundary delineation, and support more precise clinical assessment. Table 6 contains hybrid Transformer models. DS: Dice score; DSC: dice similarity coefficient; FCN/FCNs: fully convolutional network(s); HD: Hausdorff distance; MRI: magnetic resonance imaging; NMF: non-negative matrix factorization; TAG: twin attention gate; U-Net: U-shaped convolutional network.

Since the transformer model has shown superiority in modeling the global context and CNNs, along with several variants of CNNs (e.g., U-Net) outperforming local feature extraction, transformers are usually used as global feature extraction modules to refine the features extracted by CNNs, such as utilizing transformers as a global guidance module to optimize the CNN structure in the factorizer proposed by Ashtari et al. (2023). Ashtari et al. (2023) optimized the structure of CNNs and transformers via NMF and U-Net, improved the global context modeling ability of transformers and the local feature extraction ability of U-Net, and improved the accuracy of small lesion segmentation on the ISLES 2022 dataset. Wu et al. (2024) utilized transformer self-attention to optimize traditional CNNs and U-Net. The FRPNet model improved both the global context modeling ability and local feature extraction ability, enhancing the accuracy of lesion segmentation and edge detail delineation. Experimental results on several stroke datasets revealed that the proposed FRPNet model achieved better segmentation performance than U-Net, the transformer, and other attention-enhanced networks did.

In summary, transformer-based methods for medical image segmentation offer notable advantages, including superior global context modeling and the ability to capture long-range dependencies, which make them valuable for ischemic stroke lesion segmentation. However, these methods still face certain challenges, such as high computational cost, large parameter sizes, and the need for extensive training data (Malik et al., 2024). Pure transformer architectures demonstrate strong performance in capturing spatial relationships but are often computationally intensive. In contrast, hybrid architectures that combine transformers with CNN or U-Net backbones can balance global context modeling with efficient local feature extraction, leading to competitive or superior segmentation accuracy while mitigating complexity to some extent. Future research should focus on developing lightweight transformer variants, optimizing attention mechanisms, and leveraging multimodal integration to further improve performance and reduce computational demands, thereby increasing their clinical applicability in ischemic stroke lesion segmentation (Solimana et al., 2024).

#### Medical image segmentation methods based on hybrid models

Although the improved deep learning models discussed above have achieved notable advancements in medical image segmentation, research on hybrid models is still necessary when they are applied to structurally complex and significantly variable medical images. As displayed in **Additional Table 6**, hybrid models can benefit from other architectures, which will improve model interpretability and robustness, and further enhance segmentation accuracy and consistency. Kumar et al. (2020) merged self-similar fractal networks with U-Net to improve multiscale modeling and fine structure preservation, which greatly improved the Dice score, accuracy, sensitivity, and precision on the ISLES dataset. To improve segmentation efficiency and accuracy for complex structures, Wei et al. proposed an architecture named SGD-Net, which combines binary classification models with U-Net. Subsequently, Wei’s group developed an extended version of SGD-Net, called SGD-Net Plus, which can not only help achieve automatic lesion segmentation in MR images but also enable quantitative lesion analysis (Wei et al., 2022). To enhance the robustness, interpretability, and inference capability of the model, Mojiri Forooshani et al. (2022) proposed combining Bayesian inference with 3D CNNs. The proposed method achieved remarkable performance on the test dataset: the Dice score was (0.89 ± 0.08), and the modified Hausdorff distance was (2.98 ± 4.40) mm, indicating high accuracy and precision of the segmentation.

**Additional Table 6:** Summary of hybrid models applied for lesion segmentation in ischemic stroke

Studies	Models	Innovative aspects	Research results	Practical significance
Kumar et al., 2020	U-Net + self-similar fractal networks	Hybrid training strategy and self-similar (fractal) U-Net models	DS: 0.83; Precision: 0.79; Recall: 0.89	CSNet, combining fractal and U-Net architectures, improves acute stroke lesion segmentation accuracy.
Wei et al., 2022	U-Net + the second model	Semantic segmentation	DS (small lesions): 0.761; DS (large lesions): 0.830	SGD-Net and SGD-Net Plus enable accurate AIS lesion segmentation, classification, and mapping for clinical use.
Mojiri Forooshani et al., 2022	3D CNNs + Bayesian inference	Segmenting white matter hyperintensities	DSC: 0.89; HD: 2.98 mm	Bayesian 3D U-Net enables accurate and robust WMH segmentation.
Ahmed et al., 2024	Transformer + convolutional deep learning	Small stroke lesions	DSC: 0.82; Precision: 0.80; HD: 17.86 mm	A transformer with ecological augmentation improves the accuracy and robustness of chronic stroke lesion segmentation.
Wu et al., 2023b	Transformer + CNNs + BDM	The fuzzy boundary for lesion segmentation	DSC: 0.856; Precision: 0.883	W-Net with multi-scale Transformers and CNNs refines lesion boundaries and improves feature extraction.
Soh and Rajapakse, 2023	Transformer + U-Net + self- supervised	Single-modality and multimodality images	DS: 0.737; Precision: 0.825	HUT combining UNet and Transformers improves brain lesion segmentation accuracy and generalization across datasets.
Oh and An, 2024	Transformer + U-Net	Transformer computed with shifted windows	DS: 0.575; Accuracy: 0.984	Swin UNETR enables precise infarct segmentation and classification across brain regions.
Alshehri and Muhammad, 2024	Transformer + CNN	Parallel fusion and serial fusion	DSC: 0.760	A Transformer-CNN with few-shot learning improves ischemic stroke segmentation with limited data.
Nouman et al., 2024	U-Net + SwinUNETR	Feature fusion and segmentation synthesis	DSC: 0.730	Neuro-TransUNet sets a new benchmark in segmentation.
Sinha et al., 2024	U-Net + Genesis-k blocks + dual attention mechanism	Detecting fine lesions accurately	DS (DWI): 0.842; DS (FLAIR): 0.897; Accuracy (DWI): 0.972; Accuracy (FLAIR): 0.962	EnigmaNet with dual attention and wFTD loss achieves robust and accurate ischemic stroke lesion segmentation.

This table summarizes hybrid deep learning approaches for ischemic stroke lesion segmentation. These methods combine complementary strengths of different architectures to improve segmentation accuracy and enhance robustness across modalities for clinical decision-making. AIS: Acute ischemic stroke; BDM: boundary deformation module; CNNs: convolutional neural networks; CSNet: classifier-segmenter network; DS: dice score; DSC: dice similarity coefficient; HD: Hausdorff distance; HUT: Hybrid UNet and Transformer; MRI: magnetic resonance imaging; SGD-Net: semantic segmentation guided detector network; Swin UNETR: Swin Transformer-based UNetR; wFTD: weighted focal-tversky-dice loss; WMH: white matter hyperintensities.

In addition to the above studies, transformers combined with CNNs have also been utilized to overcome the weaknesses of long-range dependencies and the global context in CNNs and U-Net, while maintaining the local feature extraction advantages of CNNs and utilizing the global context modeling advantages of transformers. For example, to improve the segmentation accuracy, robustness, and runtime of the model, Ahmed et al. (2024) utilized transformers combined with CNNs, which made full use of the global modeling ability of transformers and the local feature extraction ability of CNNs, achieving an excellent Dice coefficient of 0.82 ± 0.39 on the ATLAS 2022 dataset. Wu et al. (2023b) further integrated the advantages of multiscale transformers and CNNs to propose the W-Net architecture. The proposed network uses the boundary deformation module and boundary constraint module to refine the lesion boundaries, and takes advantage of the self-attention mechanism with convolutional layers to extract more features (Wu et al., 2023b). Although the models based on the combination of transformers and CNNs have many advantages, researchers have attempted to incorporate the transformer into U-Net to improve the segmentation accuracy to achieve high-precision segmentation, such as small lesions and blurred boundary segmentation. The HUT network was proposed by Soh and Rajapakse (2023). Sinha et al. (2024) combined the Genesis-k module and dual attention mechanisms with U-Net. The proposed method achieved Dice score improvements of 41%, 32%, and 10%, respectively, over the FCN-8, U-Net, and attention U-Net models on the ISLES 2015 dataset. The proposed method not only showed better accuracy for small lesion detection but was also applicable for lesions of different sizes, shapes, and locations.

In summary, hybrid model-based methods have demonstrated potential for ischemic stroke lesion segmentation. However, challenges remain in current work, such as improving computational efficiency for large-scale applications and addressing generalization for different devices and datasets. These challenges can potentially be addressed in the future by developing methods such as self-supervised learning and reinforcement learning.

### Object detection in ischemic stroke magnetic resonance images via deep learning

Object detection is a technique used to identify, localize, and classify specific objects within medical images, aiming to determine both the category and location of the target in images. With the advancement of AI technologies, traditional object detection algorithms, which rely on manual feature extraction, suffer from poor robustness, low computational efficiency, and difficulty in handling the complexity of medical images, thus failing to meet the growing demand for efficient and accurate detection in modern medicine (Yuan et al., 2024). To address the limitations of traditional object detection algorithms, researchers have progressively applied deep learning methods to medical image target detection tasks. Deep learning leverages multilayer neural networks to automatically extract features, reducing reliance on manual input and significantly enhancing both detection accuracy and speed. Compared with image segmentation, research on object detection is still in a relatively preliminary stage, primarily due to higher computational complexity, more complex task requirements, and greater difficulty in data annotation (Fernandes et al., 2024a). As various networks have been detailed in the image segmentation section, this section aims to review the application of one-stage and two-stage CNN-based object detection methods in ischemic stroke MR images and provide a comparative analysis of the accuracy of different detection algorithms.

#### One-stage object detection

Single-stage object detection methods transform object localization and classification tasks into regression problems, thereby avoiding the process of generating candidate regions and significantly improving computational efficiency. These methods typically accomplish localization and classification in a single forward pass, thereby offering faster computation speed and making them suitable for real-time object detection tasks. You Only Look Once (YOLO) and single shot multibox detector (SSD) are two common one-stage object detection methods. YOLO, first proposed by Professor Redmon, directly predicts both bounding boxes and object classes from the entire image (Redmon et al., 2016). Although the introduction of YOLO revolutionizes object detection technology, the initial YOLOv1 exhibits poor localization accuracy and struggled with detecting small objects. YOLOv3 further enhances the detection of small and multiscale objects by introducing a three-level detection layer similar to the feature pyramid network. As shown in **Additional Table 7**, Zhang et al. (2021b) applied YOLOv3 to the automatic detection of stroke lesions in MR images and reported that its multiscale detection mechanism effectively identified the precise locations and boundaries of small lesions. Ayesha et al. (2024) subsequently applied the improved YOLOv3 to lesion detection and classification in multimodal MR images of ischemic stroke, enhancing its ability to handle small objects and complex background images through optimization of the network architecture. Subsequently, YOLOv5, which uses the PyTorch framework, has been enhanced through modifications to the loss function, convolutional modules, and data augmentation techniques, resulting in improvements in performance, usability, and scalability. Chen et al. (2022) proposed an automated ischemic stroke lesion detection method based on YOLOv5, which improves detection performance through the integration of an aggregation pooling module and an inverse attention module, achieving a positive detection rate of 0.985 across 1681 MR images of patients with stroke. Building on this idea, Chen et al. (2023a) combined multiscale features, a transformer, and channel attention mechanisms with YOLOv5 to propose the MSA-YOLOv5 model. The model demonstrated outstanding performance on the well-partitioned ISLES 2022 dataset, particularly in small lesion detection and image artifact handling.

**Additional Table 7:** Summary of one-stage object detection methods used for object detection in ischemic stroke

Studies	Models	Innovative aspects	Research results	Practical significance
Zhang et al., 2021	YOLOv3	Small object detection	mAP: 0.749; FPS: 30 s	YOLOv3 enables accurate ischemic stroke lesion detection from brain MRI.
Ayesha et al., 2024	Modified YOLOv3	Taking on information of multi-facet features	Accuracy (DWI): 0.884; Accuracy (T1WI): 0.802; Accuracy (FLAIR): 0.884	YOLOv3 with handcrafted features improves ischemic stroke lesion detection and classification on MRI.
Chen et al., 2022	TE-YOLOv5	Tracing the edge of the stroke lesion	mAP: 0.807; Precision: 0.816	TE-YOLOv5 with AP and RA modules improves the detection of small and blurred stroke lesions in DWI.
Chen et al., 2023a	MSA-YOLOv5	Small lesion size and blurred borders	mAP: 0.809; Precision: 0.829	MSA-YOLOv5 enhances AIS lesion detection, especially for small and blurred lesions.
Aljarallah et al., 2023	YOLOv7	Image enhancement, feature extraction and fine-tuned detection	Accuracy: 0.983; Precision: 0.976; Recall: 0.854	ResNest with lightweight preprocessing achieves superior AIS detection on MRI and CT with limited resources.
Elhanashi et al., 2024a	YOLOv8n	Deep learning and federated learning	Not available	YOLOv8 with federated learning enables accurate, privacy-preserving, real-time stroke detection.
Zhang et al., 2021	SSD	Predicting from shallow to deep layers	mAP: 0.898; FPS: 27.5 s	SSD enables automated ischemic stroke lesion detection from MRI with high precision.
Ayesha et al., 2024	SSD	Numerous lesions of various types, shapes, and sizes	Accuracy (DWI): 0.912; Accuracy (T1WI): 0.808; Accuracy (FLAIR): 0.908	SSD and handcrafted feature fusion with classifiers enables accurate automated stroke lesion detection.
Sailaja and Pattani, 2023	SSD + YOLOv5	Reducing the effort and time required for screening and analyzing	mAP: 0.964; Precision: 0.986; Recall: 0.985	Deep-learning models (SSD, YOLOv5) enable accurate and efficient ischemic stroke lesion detection across MRI, CT, and X-ray images.

This table summarizes representative one-stage object detection methods for ischemic stroke, focusing on model architectures, target lesion characteristics, and detection performance. These methods aim to achieve rapid and accurate lesion localization, supporting automated screening, reducing analysis time, and facilitating timely clinical decision-making. FPS: Frames per second; HD: Hausdorff distance; mAP: mean average precision; MRI: magnetic resonance imaging; SSD: Single Shot MultiBox Detector; YOLO: You Only Look Once (including versions YOLOv3, YOLOv5, YOLOv7, YOLOv8).

Although YOLOv5 is a fast and accurate object detection model, continuously updating the YOLO series is still important because of rapid technology development, application demand, and constant improvement from researchers. Aljarallah et al. (2023) used YOLOv7 for feature extraction, used a generative adversarial network for image colorization, and fine-tuned the model via the Aquila optimization algorithm, achieving an average accuracy of 0.983 and an F1 score of 0.973 on an ischemic stroke MRI dataset. YOLOv8 is developed with a new backbone, improved loss function, and regularization technique in the model, which makes YOLOv8 a lightweight model and enhances its deployment ability, inference speed and training efficiency. Elhanashi et al. (2024b) used YOLOv8n as the main architecture of their model and applied a federated learning approach to develop an efficient and privacy-preserving real-time stroke detection system. Elhanashi et al. (2024a) also applied the YOLOv8 model to extract subtle information related to stroke and successfully differentiated stroke and nonstroke real-time cases with accurate results while preserving data privacy via the federated learning approach.

The SSD model, proposed by Professor Liu in 2016, utilizes feature maps at multiple scales and the design of default boxes to enhance the detection capability for small and multiscale objects (Liu et al., 2016). Zhang et al. (2021b) employed SSD to independently predict from shallow to deep layers, integrating multilevel information for object detection. Compared with YOLOv3, SSD demonstrated superior accuracy in detecting ischemic stroke lesions across 5668 brain MR images. Ayesha et al. (2024) reported that SSD, which leverages multiscale feature maps, anchor box mechanisms, and end-to-end training methods, demonstrated outstanding performance in lesion detection and classification in multimodal MR images of ischemic stroke. Furthermore, Sailaja and Pattani (2023) proposed an object detection method that combines SSD and YOLOv5, leveraging the advantages of SSD in multilevel information processing and the strengths of YOLOv5 in small lesion detection.

In summary, a one-stage object detection method directly detects objects in an image by classifying objects and regressing the bounding box in a single network and has the following advantages: high computational efficiency and fast inference. Compared with other models, the YOLO model has been updated and improved many times. It can be used to detect small lesions in ischemic stroke patients with powerful feature extraction and accurate bounding box localization. The SSD algorithm uses a multiple-layer feature map design and can detect lesions of different sizes. However, these methods still have some limitations in terms of the MRI background, complex background, and overlapping lesions. In the future, we will explore more efficient network architectures and optimization algorithms to improve the efficiency and accuracy of models.

#### Two-stage object detection

Owing to the advantages of speed and efficiency, one-stage object detection methods may encounter problems such as false positives, missed detections, and/or misdetections on more complex medical images. Two-stage object detection methods first generate candidate regions and then localize and classify objects within these regions. It is more suitable for more complex applications or more precise requirements. The representative two-stage object detection methods are the region-based CNN (R-CNN), fast region-based CNN (Fast R-CNN), and faster region-based CNN (Faster R-CNN). The R-CNN, proposed by Professor Girshick in 2014, fuses CNNs and selective search algorithms, offering a new approach to object detection based on the basis of region proposals (Girshick et al., 2014). As shown in **Additional Table 8**, Ayesha et al. (2024) used an R-CNN object detection network on an MRI database to automatically detect ischemic stroke lesions. Sun et al. (2023) incorporated low-rank decomposition algorithms based on CNNs into the R-CNN model design for ischemic stroke lesion segmentation and localization. Although the R-CNN can accurately detect the location of the lesion, its training process is multistage, and there is redundant feature computation, which makes its training speed and inference speed slower.

**Additional Table 8:** Summary of two-stage object detection methods used for object detection in ischemic stroke

Studies	Models	Innovative aspects	Research results	Practical significance
Ayesha et al., 2024	R-CNN	Utilizing ResNet101 for the extraction of features	Accuracy (DWI): 0.882; Accuracy (T1WI): 0.790; Accuracy (FLAIR): 0.902	R-CNN and handcrafted features enable accurate automated ischemic stroke lesion detection from multimodal MRI.
Sun et al., 2023	R-CNN	CNNs and a low-rank decomposition algorithm	Accuracy: 0.78; Sensitivity: 0.85	LTRCNN enables accurate AIS lesion segmentation.
Zhang et al., 2021	Faster R-CNN	Using ResNet-101 for feature extraction	mAP: 0.765	Faster R-CNN enables accurate automated ischemic stroke lesion detection from MRI.
Saad et al., 2024	Faster R-CNN	Detecting the hypointense and hyperintense lesions	Accuracy: 0.646; Sensitivity: 0.646	Faster R-CNN enables automated classification of stroke subtypes.
Daoudi et al., 2020	Mask R-CNN	Resolving instance-level recognition tasks	mAP: 0.81	Mask R-CNN enables effective detection and segmentation of stroke lesions in multimodal MRI with high accuracy.
Wei et al., 2022	SGD-Net Plus	Semantic segmentation and object detection	Accuracy: 0.956; Sensitivity: 0.958	SGD-Net Plus enables precise AIS lesion segmentation and classification on MRI.

This table summarizes representative two-stage object detection methods for ischemic stroke, focusing on model architectures, feature extraction strategies, and detection tasks. These methods aim to improve lesion detection accuracy and sensitivity, enabling precise localization and characterization of stroke lesions to support timely diagnosis and treatment planning. AIS: Acute ischemic stroke; CNN: convolutional neural network; mAP: mean average precision; Mask R-CNN: mask region-based convolutional neural network; MRI: magnetic resonance imaging; R-CNN: region-based convolutional neural network; SGD-Net: semantic segmentation guided detector network.

To decrease the computational complexity and increase the detection speed, Professor Girshick proposed the Fast R-CNN model (Girshick, 2015). Compared with the traditional model, Fast R-CNN can increase the detection speed and reduce the computational complexity by utilizing Roi pooling and an end-to-end training method (Girshick, 2015). Even though the Fast R-CNN was an improvement over the original R-CNN, it still relied on a selective search to generate candidate regions, leading to a large amount of computational overhead and a slower processing speed, which made it unsuitable for real-time detection. Ren et al. (2015) proposed Faster R-CNN to solve this problem. Region proposal networks (RPNs) in Faster R-CNN are connected with the feature extraction process, and they can be trained end-to-end to improve both the detection speed and efficiency (Ren et al., 2017). Faster R-CNN was used by Zhang et al. (2021b) in the automated detection of ischemic stroke lesions on MR images, and the average precision achieved was 0.765. According to the study by Saad et al. (2024), Faster R-CNN with RPN instead of selective search was connected with CNNs, resulting in improved detection performance on public and clinical datasets.

The above methods may have some limitations in dealing with complex-shaped, possibly overlapping stroke lesions, especially for multiple high-resolution lesion detection tasks. As an extension of R-CNN, Kiernan et al. (2024) proposed the Mask R-CNN model by adding instance segmentation at the pixel level with precision. The model speeds up the feature extraction process by sharing convolutional features over the image and uses RPNs to generate candidate regions on the fly, which greatly improves the detection performance on complex medical images. Unlike the above methods, Daoudi et al. (2020) utilized Mask R-CNN for stroke lesion detection and segmentation. They combined RPN, CNNs, and segmentation networks, which could achieve pixel-level segmentation and accurate localization of stroke lesions with a mAP of 0.81 for different sizes of lesions. In some scenarios of two task joint detection, two-stage methods have advantages in handling multiple tasks. For example, Wei et al. (2022) used the SGD-Net Plus model, which combines a medical image semantic segmentation task and an object detection task in the model. They reported that the model could accurately localize ischemic stroke lesions and quantify the sizes of different lesions in brain regions.

In summary, two-stage object detection methods have obvious advantages in handling complex-shaped lesions and possible overlaps and in localizing lesions. The two-stage method with candidate region generation and refinement can greatly improve the detection performance for complex lesions. However, two-stage object detection methods also have several limitations, such as high computational complexity, long training times, and poor quality of candidate region generation. Therefore, reducing computational complexity and improving the quality of candidate region generation are important future research directions for obtaining better two-stage object detection methods in complex medical applications.

### Image classification in ischemic stroke magnetic resonance images via deep learning

Image classification is one of the most basic applications of deep learning in medical image analysis; it is used mainly to support disease diagnosis and classification, and few works have applied deep learning to evaluate disease severity (Chen et al., 2025a). In the early stage, most medical image classification methods used traditional machine learning algorithms, such as support vector machines (SVMs), k-nearest neighbors, and random forests (Ignatenko et al., 2024; Liang et al., 2024; Khan et al., 2025b). However, these methods are heavily dependent on handcrafted features and require large volumes of high-quality images for training, which limits their robustness in complex clinical scenarios characterized by organ variability and lesion heterogeneity. In recent years, an increasing number of works have applied deep learning methods for medical image classification to address the limitations of traditional machine learning algorithms. Compared with traditional machine learning algorithms, deep learning methods can automatically learn features from multilayer neural network architectures and optimize the whole classification procedure in an end-to-end way, which has been proven to be more accurate and more robust in analyzing multidimensional and complex medical images (Shastry et al., 2022). Unlike image segmentation and object detection tasks, image classification usually extracts global or regional features from the whole image or a certain area of interest and outputs the final classification decision through a classifier. In this section, we review recent advances in ischemic stroke image classification via commonly used deep learning models, such as CNNs, transformer-based architectures, and hybrid models. Additionally, the limitations of these models in ischemic stroke image classification are discussed, and future research directions are proposed.

#### Medical image classification methods based on convolutional neural network architectures

As a typical architecture in the image processing field, CNNs can automatically extract multiscale spatial features from multidimensional data and handle them efficiently, which avoids a time-consuming feature engineering process and is widely used in brain MR image classification tasks. As shown in **Additional Table 9**, Polson et al. (2022) proposed the use of a deep learning tool based on CNNs for automatically classifying time since stroke (TSS) without expert interpretation via MRI data to indicate whether the stroke occurred within 4.5 hours. The model obtained an accuracy of 0.724 and a sensitivity of 0.757 on an external validation set and outperformed the sensitivity of human experts (Polson et al., 2022). Zhang et al. (2021a) used both 2D and 3D CNNs combined with adaptive transfer learning strategies, achieving an area under the curve (AUC) of 0.74, a sensitivity of 0.70, a specificity of 0.81, and an accuracy of 0.758 for stroke onset within the 4.5-hour therapeutic window. To further improve the accuracy of TSS classification and reduce the cross-stage error of training and validation, Zhang et al. (2025a) proposed a synchronous dual-stage network (SDS-Net), in which the lesion localization and TSS classification tasks of acute ischemic stroke patients are trained and optimized simultaneously in a unified network. The feature-sharing mechanism further improved the model’s ability to integrate temporal-spatial information and achieved an AUC of 0.879 and an accuracy of 0.800 on an independent external test set (Zhang et al., 2025a).

**Additional Table 9:** Summary of CNN methods applied for image classification in ischemic stroke

Studies	Models	Innovative aspects	Research results	Practical significance
Cetinoglu et al., 2021	CNNs	A transfer learning approach based on MobileNetV2 and EfficientNet-B0 CNNs	Accuracy (MobileNetV2): 0.96; Accuracy (EfficientNet-B0): 0.93	MobileNetV2 and EfficientNet-B0 enable accurate stroke and vascular classification from DWI.
Zhang et al., 2021	2D CNNs 3D CNNs	An intra-domain task-adaptive transfer learning method	Specificity: 0.81; Sensitivity: 0.70; AUC: 0.74	2D and 3D CNNs with transfer learning enable accurate stroke onset detection within the 4.5-hour therapeutic window.
Lu et al., 2021	Patch-based CNNs	Pretrained CNNs and patch-based CNNs	F1 score: 0.895; AUC: 0.881	Gated attention CNNs improve lesion classification from multimodal MRI, achieving high accuracy in AIS detection.
Polson et al., 2022	CNNs	Achieving higher generalization performance	Accuracy: 0.724; Sensitivity: 0.757	CNN-based models enable automatic TSS classification from MRI within 4.5 hours, outperforming human expert sensitivity.
Wei et al., 2022	U-Net	Semantic segmentation guided detector network	Accuracy (size): 0.956; Accuracy (location): 0.93	The two-stage SGD-Net improves AIS lesion size and location classification accuracy over single-stage models.
Oura et al., 2024	CNNs	The montage image	Accuracy: 0.76; AUC: 0.94	Montage multimodal MRI with CNNs improves AIS classification accuracy and AUC over pseudo-color images.
Zhang et al., 2025	CNNs	Infarct voxel identification and TSS classification	Accuracy: 0.844; AUC: 0.914	SDS-Net jointly performs lesion localization and TSS classification, enhancing temporal-spatial integration and achieving high accuracy.
Felehgari et al., 2025	CNNs	Adding CNNs Layer-ResNet-50 and CNNs Layer-MobileNetVl	Accuracy: 0.98 Sensitivity: 0.99 AUC: 0.99	CNNs with transfer learning enable accurate stroke type classification and NIHSS severity prediction.
Kim et al., 2025	CNNs	Convolutional neural network- based classifiers	AUC (internal test): 0.934 AUC (external): 0.913	A CNN-based stroke classification model achieved high internal and external AUCs.

This table summarizes representative CNN-based methods for ischemic stroke image classification. These methods aim to improve classification accuracy, enhance model generalization, and support rapid and reliable stroke subtype identification for clinical decision-making. AIS: Acute ischemic stroke; AUC: area under the curve; CNNs: convolutional neural networks; MRI: magnetic resonance imaging; NIHSS: National Institutes of Health Stroke Scale; TSS: time since stroke; U-Net: U-shaped convolutional network.

Although the classic CNN architectures are very successful in capturing local features, they have several limitations, such as the inability to capture critical features and the small sample size problem in medical image classification tasks. To overcome these limitations, other techniques have been added to the model, such as attention methods and transfer learning, which have greatly improved the classification performance on various classification tasks. To enhance the ability of the model to capture critical features even more, Lu et al. (2021) designed and validated a gated attention mechanism-enhanced CNN model for the classification of lesions from multimodal MR images. The method achieved remarkable performance in the classification of acute ischemic stroke, with an AUC of 0.881 and an F1 score of 0.895. Later, Cetinoglu et al. (2021) used transfer learning methods to develop enhanced CNN models based on MobileNetV2 and EfficientNet-B0 architectures for assessing the automated detection of strokes and classification of vascular territories from diffusion-weighted imaging (DWI) images. Subsequently, Felehgari et al. (2025) developed CNN-based ACL-MobileNetV1 and ACL-ResNet-50 models via transfer learning techniques for stroke type classification and severity assessment tasks. The results demonstrated that ACL-MobileNetV1 achieved an accuracy of 0.98 and was thus superior in distinguishing among the normal, ischemic, and hemorrhagic stroke categories. In contrast, ACL-ResNet-50 achieved the highest accuracy in predicting NIHSS score intervals from DWI images among the models studied, with an accuracy of 0.92 (Felehgari et al., 2025).

Owing to the existence of structural variability, multimodal diversity, and cross-center variability in stroke MR images, researchers have explored more efficient and generalized classification methods continuously. To enhance the ability of the model to capture complex features of the lesion, Wei et al. constructed and optimized a two-stage deep learning model named SGD-Net for the classification of lesion size and vascular territory distribution in acute ischemic stroke cases. The experimental results revealed that the accuracies of SGD-Net for the classification of lesion size ranged from 0.867–0.956, and those for the classification of lesion location ranged from 0.860–0.930, which were significantly higher than those of conventional single-stage models (Wei et al., 2022). To more effectively utilize multimodal MR information, Oura et al. (2024) aggregated four different types of MRI modalities into montage images and then applied CNNs for the classification of acute ischemic stroke patients. The accuracy and AUC of the ROC for the classification of acute ischemic stroke patients were 0.76 and 0.94, respectively, for montage images, which were significantly greater than those of conventional pseudocolor images (0.54 and 0.76) (Oura et al., 2024). To further improve the generalizability and cross-center generalization ability, Kim et al. (2025) trained a CNN-based classification model on a dataset from two stroke centers and then validated it on patient data from two different institutions. The results revealed that the AUC obtained during internal validation was 0.934, and the maximum AUC obtained during external validation was 0.913.

Typically, CNN-based image classification methods have demonstrated promising potential in ischemic stroke MRI classification tasks. However, current studies still face some challenges, such as limited data and poor model generalizability (Cui et al., 2022; Hossain et al., 2023). It is reasonable to expect that classification accuracy could be improved by using more advanced methods, such as self-supervised learning strategies and federated learning approaches, in future research to address the challenges mentioned above.

#### Medical image classification methods based on transformer and hybrid models

In recent years, transformer-based architectures and their variants have increasingly demonstrated powerful modeling capabilities in medical image analysis. Compared with CNN architectures, transformer models leverage self-attention mechanisms to capture long-range dependencies, making them particularly well-suited for learning global structural representations. As shown in **Additional Table 10**, Abbaoui et al. (2024) used the Vision transformer to classify stroke types in MR images. The model achieved an accuracy of 0.976, substantially outperforming the conventional CNN model (VGG-16) and highlighting the superior image classification capacity of transformer-based architectures. To combine the global contextual awareness of transformer architectures with the precise segmentation capabilities of U-Net, Oh and An (2024) adopted the Swin UNETR model, a hybrid framework integrating the Swin transformer and U-Net, for classifying lesion location and type in cerebral infarction. When trained and validated on MRI data from 309 patients, the model achieved accuracies of 0.984 and 0.984, respectively.

**Additional Table 10:** Summary of Transformer and hybrid models applied for image classification in ischemic stroke

Studies	Models	Innovative aspects	Research results	Practical significance
Abbaoui et al., 2024	Transformer	A vision transformer model	Accuracy: 0.976	Vision transformer significantly improves stroke type classification from MRI, surpassing conventional CNN models.
Oh and An, 2024	Transformer + U-Net	Integrating U-Net and Transformer	Accuracy (training): 0.984; Accuracy (validation): 0.984	Swin UNETR integrates Transformer and U-Net to achieve highly accurate classification of cerebral infarction lesion location and type.
Heo et al., 2020	CNNs + multi- CNNs	LSTM and MLP	AUC: 0.805	Multi-CNNs with cross-validation optimization improve AIS lesion classification robustness and accuracy.
Ye et al., 2023	OEDL method	Combining features and mixed sampling	Accuracy: 0.957; Recall: 0.948; Precision: 0.940	OEDL integrating clinical and radiomic features with hybrid sampling markedly improves stroke prognosis classification.
Koska et al., 2025	3D CNNs+ LSTM- CNNs	Developing an end-to-end deep learning model	Accuracy: 0.88; F1 score: 0.80; AUC: 0.98	Mobile-Crop enhances stroke region classification by combining CNN with cropping and augmentation strategies.

This table summarizes representative Transformer-based and hybrid deep learning methods for ischemic stroke image classification, highlighting their network architectures, feature integration strategies, and evaluation performance. These methods aim to improve classification accuracy, enhance feature representation, and support more reliable and efficient clinical decision-making. AUC: Area under the curve; CNNs: convolutional neural networks; LSTM: long short-term memory; MLP: multi-layer perceptron; MRI: magnetic resonance imaging; OEDL: optimized ensemble deep learning; U-Net: U-shaped convolutional network.

In recent years, hybrid modeling strategies have been increasingly used to improve performance in stroke MRI classification tasks. By integrating diverse deep learning architectures, such approaches have demonstrated notable improvements in model robustness, generalizability, and clinical adaptability. The following studies highlight the effectiveness of hybrid models in addressing complex classification tasks (Heo et al., 2020; Ye et al., 2023; Sun et al., 2024; Koska et al., 2025). To improve the robustness of deep learning models, Heo et al. (2020) integrated multiple deep neural network architectures, including CNNs, multi-CNN, long short-term memory (LSTM), and multilayer perceptron, and used cross-validation together with grid search to optimize model performance. The results showed that the multi-CNN architecture achieved the best performance on MRI data from patients with acute ischemic stroke, with an AUC of 0.805 (Heo et al., 2020). To improve adaptability to data heterogeneity, Ye et al. (2023) proposed an optimized ensemble deep learning approach. This method integrates clinical and radiomic features and incorporates a hybrid sampling strategy, resulting in a significant enhancement in stroke prognosis classification performance (Ye et al., 2023). To enable high-precision automated classification of complex regions, Koska et al. (2025) developed an end-to-end hybrid deep learning model that integrates 3D CNNs, attention mechanisms, LSTM-CNNs, and a slice-level parallel encoding architecture for classifying brain regions in patients with stroke on the basis of DWI images. With the incorporation of clinical domain knowledge, the accuracy of the model further increased to 0.93, achieving performance comparable to that of experts (Koska et al., 2025).

In this context, transformer-based architectures, as well as hybrid models, have shown promising performance for the classification of ischemic stroke via MRI sequences. However, challenges still hamper current studies, such as the lack of model robustness to noise and imaging artifacts, the increased vulnerability of classification bias due to the data imbalance problem, and the lack of interpretability of model decisions (Boone et al., 2022; Tran et al., 2025). In this regard, we believe that future research should focus on addressing the above challenges by incorporating multimodal data for better feature representation, applying data augmentation and transfer learning to address sample imbalance, and integrating explainable AI to enhance model interpretability.

Overall, the focal lesion profile of ischemic stroke makes lesion-centric architectures such as U-Net, nnU-Net, or hybrid frameworks incorporating ROI priors particularly well suited for segmentation and detection tasks while also supporting classification objectives such as stroke subtype identification and therapeutic time window estimation. These method-disease alignments illustrate how task selection and network design can be tailored to disease-specific imaging characteristics, providing a methodological reference for extending deep learning applications to other neurodegenerative disorders. Future research will likely benefit from self-supervised learning, diffusion-driven data augmentation, and few-shot or weakly supervised approaches, which can alleviate data scarcity and variability while enhancing the robustness and translational potential of stroke imaging analysis. The following sections examine Alzheimer’s disease and Parkinson’s disease as representative conditions to explore deep learning strategies for etiology-specific cognitive impairment systematically.

## Deep Learning Methods for Brain Magnetic Resonance Imaging Analysis in Alzheimer’s Disease

Although various forms of cognitive impairment exhibit overlapping clinical manifestations, they differ substantially in their underlying pathophysiological mechanisms and affected brain regions. To gain a deeper understanding of the variability in brain region recognition, cross-disease adaptability, and the generalization capacity of deep learning models across distinct diseases, this review also incorporates representative neurodegenerative diseases, such as Alzheimer’s disease, for which cognitive impairment is a hallmark feature. Alzheimer’s disease is the most prevalent neurodegenerative disorder characterized by cognitive impairment (Couvy-Duchesne et al., 2025). In its early stages, it commonly presents with memory deterioration, followed by progressive impairments in executive function, spatial orientation, and language abilities as the disease progresses. Neuroimaging studies have revealed pronounced structural atrophy and weakened functional connectivity in several brain structures in patients with Alzheimer’s disease, particularly in the hippocampus, entorhinal cortex, amygdala, and inferior parietal lobule (Yen et al., 2023).

In recent years, deep learning has shown notable advantages in tasks related to Alzheimer’s disease imaging owing to its ability to automatically extract multiscale structural features from high-dimensional data, model complex nonlinear relationships, and integrate multimodal information. In ischemic stroke research, deep learning models are predominantly applied to three core tasks: lesion segmentation, object detection, and image classification. However, in Alzheimer’s disease studies, where imaging features are characterized by diffuse brain atrophy and a lack of clearly demarcated focal lesions, the use of object detection remains limited (Hammers et al., 2025). Therefore, this section highlights deep learning-based approaches to lesion segmentation and image classification in Alzheimer’s disease imaging, with a focus on their methodological progression and task-specific challenges.

### Lesion segmentation in Alzheimer’s disease magnetic resonance images via deep learning

Lesion segmentation, as a fundamental task in medical image analysis, plays a pivotal role in Alzheimer’s disease research. Alzheimer’s disease is commonly characterized by progressive brain atrophy, with especially prominent volume loss in structures such as the hippocampus and entorhinal cortex. Image segmentation enables researchers to precisely delineate key brain regions from MRI and other neuroimaging modalities, facilitating quantitative analysis of structural boundaries and volumetric changes. Traditional segmentation methods often fail to achieve satisfactory accuracy when applied to MRI scans of Alzheimer’s disease patients because of challenges posed by brain atrophy patterns, reduced tissue contrast, and blurred anatomical boundaries (Sghirripa et al., 2025). In recent years, deep learning-based image segmentation methods, particularly those employing CNNs, U-Net, and hybrid models, have demonstrated significant advantages in the automated segmentation of Alzheimer’s disease-related brain regions because of their superior feature extraction and spatial information preservation capabilities.

As shown in **Additional Table 11**, Liu et al. (2020) used multitask CNNs to perform automated segmentation of the hippocampus in MR images, achieving a Dice similarity coefficient of 0.87 on the ADNI dataset. This work provides a foundation for subsequent improvements and optimization of segmentation models. Subsequently, Jin et al. (2024) applied an enhanced convolutional architecture, 3D DenseNet, to structural segmentation of brain MR images and integrated a global attention module into the network to improve its capacity for feature representation, particularly in regions such as the hippocampus and cortical areas (Jin et al., 2024). To further improve the capacity of the model for capturing multiscale information, Muksimova et al. (2025) proposed a hybrid approach that integrates densely connected convolutional networks with attention mechanisms. Their method achieved high segmentation accuracy and favorable computational efficiency on the ADNI dataset. In terms of model optimization strategies, Chen et al. (2023b) proposed a novel deep learning model, RBS-Net, for automatic segmentation of the hippocampal region in MR images. The model introduces a distance map supervision mechanism and a structural similarity learning task to jointly optimize boundary delineation and anatomical structure representation, achieving an average Dice coefficient of 0.898 on the HarP dataset and outperforming several mainstream methods under limited-sample conditions. In the domain of structural segmentation optimization, Rao et al. (2025) proposed a brain region segmentation network based on the ResUNet architecture to extract anatomical structures from the MR images of patients with Alzheimer’s disease. By integrating residual connections with an encoder-decoder framework, the model exhibits strong robustness in complex scenarios involving boundary ambiguity and poorly defined small structures (Rao et al., 2025). For fine-grained anatomical segmentation, Maity et al. (2024) used deep-residual U-Net and DeepLabV3^+^ models to segment the hippocampal and ventricular regions in fMRI images. DeepLabV3^+^ achieved superior performance, thereby validating its effectiveness in delineating complex anatomical structures (Maity et al., 2024).

**Additional Table 11:** Summary of deep learning models used in Alzheimer's disease

Studies	Models	Innovative aspects	Research results	Practical significance
Liu and Yan, 2020	DBN	Semi-automatic model	DS: 0.84	A hybrid model combining DBN priors with contour refinement enables accurate hippocampal segmentation in MRI.
Liu et al., 2020	CNNs 3D DenseNet	Segmentation results for the classification task	DS: 0.87; Accuracy: 0.889; AUC: 0.925	Multi-task CNNs achieve accurate hippocampal segmentation in MRI, supporting AD diagnosis.
Irie et al., 2020	3D CNNs	Residual extraction	Accuracy: 0.90	Residual 3D CNNs enable accurate classification of AD, iNPH, and healthy controls.
Ocasio and Duong, 2021	3D CNNs	Transfer learning	Accuracy (AD *vs*. NC): 0.887	Sequential CNN with zero-shot transfer learning improves MRI-based prediction of MCI-to-AD conversion.
Mehmood et al., 2021	VGG	Layer-wise transfer learning	Accuracy (AD *vs*. NC): 0.987	Layer-wise transfer learning with VGG improves multi-class classification of NC, MCI, and AD.
Wang et al., 2022b	SAE	Multi-atlas brain segmentation	Accuracy: 0.96 to 1; AUC: 0.97 to 1	Stacked autoencoder integrating MRI features with metabolite information enhances AD classification performance.
Tuan et al., 2022	GMM SVM XGBoost CNNs	Segmentation for the classification	Accuracy (86): 0.88 Accuracy (126): 0.80	Gaussian mixture models combined with CNN improve brain tissue segmentation in AD MRI.
Cobbinah et al., 2022	CAAECRAT	Two-way classification tasks	Accuracy (AD *vs*. HC): 0.918	A two-stage framework with an adversarial autoencoder and residual attention network enhances cross-site MRI classification of AD, MCI, and NC.
Herzog and Magoulas, 2022	CNNs SVM LDA KNN	Multiple modules	Accuracy (AD *vs*. HC): 0.901	An eight-layer CNN with an ImageNet-pretrained backbone and multiple classifiers enhances discrimination between AD and HC.
Park et al., 2023	TabNet	Interpretable network	AUC: 0.951	The interpretable deep model TabNet enhances classification performance between AD and NC.
Raza et al., 2023	CNNs	Through a transfer learning approach	Accuracy: 0.978	Gray matter extraction combined with transfer learning in CNNs enhances AD classification performance.
Chen et al., 2023b	RBS-Net	Boundary segmentation	DS: 0.898	RBS-Net improves hippocampal segmentation under limited-sample conditions.
Khan et al., 2022	VGG	Transfer learning	Accuracy: 0.979	Transfer learning with gray matter extraction and layer-wise freezing enhances the classification of AD in small-sample scenarios.
Zheng et al., 2023	HGM-cNet	Two identical CNNs	DS > 0.89	HGM-cNet leverages gray matter probability maps with attention and residual modules to improve bilateral hippocampal segmentation.
Zhang et al., 2023	3DRA-Net	3D residual attention network	AUC (ADNI): 0.95	The 3D residual attention network enhances AD vs. NC classification and shows robust generalizability across cohorts.
Balasundaram et al., 2023	CNNs ResNet50	Vision transformer	Accuracy: 0.976	Localized hippocampal feature extraction improves training efficiency while maintaining the accuracy of classification of AD severity.
Jin et al., 2024	DenseNet	MobileNetV3	Accuracy (AD vs. NC): 0.979	3D DenseNet with global attention improves MRI segmentation of hippocampal and cortical regions.
Gao et al., 2024	GeoLongSeg	Two-stage network	Accuracy: 0.833 AUC: 0.911	GeoLongSeg integrates deep learning and geometric modeling to improve hippocampal segmentation consistency and reliability.
Nithya et al., 2024	ResNet-50	Links between residual layers	Accuracy: 0.95	The K-means and ResNet-50-based model enhances AD recognition while effectively reducing overfitting.
Odimayo et al., 2024	Sneurodcnn	Regularisation techniques	Accuracy: 0.978; Specificity: 0.97	The lightweight structure-aware CNN effectively detects localized brain atrophy, enhancing accuracy in identifying structural brain changes.
Rao et al., 2025	ResUnet	EfficientNetB7	Accuracy: 0.993; Accuracy: 0.994	The ResUNet-based model robustly segments brain structures with ambiguous boundaries and small regions.
Maity et al., 2024	Residual U-Net	DeepLabV3 VGG-16 VGG- 16-RF	Accuracy: 0.946; Accuracy: 0.969	DeepLabV3+ effectively segments hippocampal and ventricular regions, demonstrating strong capability in complex anatomical delineation.
Vinukonda and Jagadesh, 2025	DeepLabV3Enhanced ResNext	A hybrid deep learning	Accuracy: 0.981; AUC: 0.97	The enhanced DeepLabV3+ with ResNeXt enables accurate lesion segmentation and stage-wise classification of AD.
Muksimova et al., 2025	CNNs MSB	Attention mechanism	DS: 0.93; Jaccard Index: 0.88	A hybrid dense CNN with attention improves the accuracy and efficiency of AD image segmentation.
Gowsikraja et al., 2025	SBERO_Deep SNN	Segmentation and classification task	Accuracy: 0.905; Specificity: 0.902	SBERO_Deep SNN combining UNeXT segmentation and spiking neural networks enhances early AD diagnosis.

This table summarizes representative deep learning models applied in Alzheimer's disease for classification and segmentation tasks, detailing their network architectures, task focus, and evaluation metrics. These methods aim to enhance diagnostic accuracy, automate feature extraction, and support early detection and disease progression monitoring in clinical settings. AD: Alzheimer's disease; ADNI: Alzheimer's disease neuroimaging initiative; AUC: area under the curve; CAAE: convolutional adversarial autoencoder; CNNs: convolutional neural networks; CRAT: convolutional residual soft attention network; 3DRA-Net: 3D residual attention network; DBN: deep belief network; DenseNet: densely connected convolutional network; DS: dice score; GMM: gaussian mixture model; HC: healthy controls; HGM: gray matter probability maps; KNN: k-nearest neighbors; iNPH: idiopathic normal pressure hydrocephalus; LDA: linear discriminant analysis; LSTM: long short-term memory; MCI: mild cognitive impairment; MRI: magnetic resonance imaging; MSB: multi-scale block; NC: normal controls; RBS-Net: region-boundary and structure net; SAE: stacked auto-encoder neural network; SNeurodCNN: structure-aware convolutional neural network; SVM: support vector machine; TabNet: interpretable network algorithm; VGG: visual geometry group; XGBoost: extreme gradient boosting.

Although segmentation based on a single model has achieved promising performance in medical images, there are still many challenges in its application, such as the anatomical complexity of brain tissue, blurred boundaries of lesions, and variation between different datasets. These challenges cause low accuracy of segmentation, oversmoothed boundaries, missed small lesions, and poor generalization ability on different datasets. To achieve higher accuracy and better generalization ability, researchers have gradually focused on hybrid models to combine the merits of different models for more effective image segmentation. Liu and Yan (2020) applied the deep belief network method to generate subject-specific prior shapes of the hippocampal lattice Boltzmann model, and then obtained accurate segmentation of the hippocampal region in MR images through contour refinement. In the integration of geometric modeling and deep learning, Gao et al. (2024) proposed a two-stage segmentation framework, GeoLongSeg. This method first combines a 3D U-Net with an attention mechanism to perform initial segmentation and then incorporates geodesic shape regression to model hippocampal evolution and enhance longitudinal consistency. The experimental results revealed that GeoLongSeg outperformed several mainstream methods in terms of test‒retest reliability, variance ratio, and atrophy trajectory (Gao et al., 2024). Zheng et al. (2023) proposed a cascaded deep learning framework, HGM-cNet, which incorporates gray matter probability maps (HGMs). The architecture consists of two sequential CNNs, each equipped with attention modules, residual connections, and DropBlock regularization. In bilateral hippocampal segmentation tasks, HGM-cNet achieves an average Dice coefficient above 0.89 and outperforms multiple models by over 1% in accuracy. Despite demonstrating significant advantages in segmentation tasks, hybrid models continue to face challenges such as architectural complexity, high training overhead, and the absence of standardized fusion strategies (Kale and Chavan, 2025). These limitations are especially pronounced in multicenter settings, imbalanced datasets, and weakly supervised scenarios, highlighting the need for further methodological refinement and extensive validation.

In summary, lesion segmentation plays a pivotal role in the structural analysis of Alzheimer’s disease-related brain changes. Deep learning models built upon CNNs and U-Net architectures have demonstrated significant advancements in the automated delineation of critical structures such as the hippocampus and entorhinal cortex. However, owing to the inherent complexity of brain structures, the challenge of detecting small-volume lesions, ambiguous anatomical boundaries, and the limited availability of annotated data, current methods continue to face shortcomings in terms of their generalizability, overfitting resistance, and clinical adaptability (Pu et al., 2023). In recent years, hybrid models incorporating geometric priors and attention mechanisms have emerged, significantly enhancing model robustness and demonstrating strong cross-domain adaptability in multiple datasets (Chen et al., 2025b; Yan et al., 2025). Future research may further emphasize multimodal data integration, self-supervised learning strategies, lightweight model design, and improved cross-platform generalizability (Zhang et al., 2024).

### Image classification in Alzheimer’s disease magnetic resonance images via deep learning

Image classification represents a fundamental task in deep learning-based medical image analysis and plays a critical role in the auxiliary diagnosis and clinical staging of Alzheimer’s disease. In contrast to image segmentation, which targets pixel-level delineation of anatomical structures, image classification emphasizes the extraction of global or localized features from whole-brain images or key ROIs to infer disease category and severity. In recent years, with the development of deep learning models such as CNNs, the accuracy and automation of Alzheimer’s disease image classification have steadily improved. As shown in **Additional Table 11**, Balasundaram et al. (2013) investigated the classification of Alzheimer’s disease severity by comparing local and global image representations. Specifically, they extracted hippocampal regions from MR images and applied CNNs, ResNet50, and multilayer neural networks, which revealed that localized feature extraction significantly improved training efficiency without compromising classification accuracy. Herzog and Magoulas (2022) constructed a custom eight-layer CNN integrated with an ImageNet-pretrained backbone and multiple classification modules, such as Softmax, SVM, linear discriminant analysis, and k-nearest neighbors. The model achieved an accuracy of 0.901 in distinguishing patients with Alzheimer’s disease from healthy controls. Odimayo et al. (2024) proposed a lightweight, structure-aware CNN tailored for the extraction of localized brain atrophy features. The model achieved an accuracy and sensitivity of over 0.97 across multiple MRI scans, highlighting its strong discriminative ability in detecting structural brain changes.

To capture both the spatial and semantic features of complex brain structures more effectively, researchers have continuously refined and extended CNNs. Irie et al. (2020) incorporated residual feature extraction into 3D CNNs to classify Alzheimer’s disease, idiopathic normal pressure hydrocephalus, and cognitively healthy individuals, achieving a classification accuracy of 0.90. Zhang et al. (2023) developed a three-dimensional residual attention network, which achieved a classification accuracy of 0.916 for Alzheimer’s disease *versus* normal control on the ADNI dataset and demonstrated robust generalizability with an external validation accuracy of 0.892 across multiple independent cohorts. Although CNNs and their variants have achieved remarkable performance on Alzheimer’s disease image classification tasks, the accuracy of these methods is limited when the amount of labeled data is small. Recently, transfer learning has been proven to improve the training efficiency of models in small-sample scenarios. Ocasio and Duong (2021) used three CNNs in combination with two transfer learning strategies to construct an MRI-based risk prediction model for the conversion of MCI to Alzheimer’s disease. A sequential CNN combined with zero-shot transfer learning achieved an accuracy of 0.793, demonstrating the advantages of the transfer learning approach in disease forecasting (Ocasio and Duong, 2021). Raza et al. (2023) used gray matter extraction as a preprocessing step and transfer learning in CNNs, and achieved a maximum classification accuracy of 0.978 for Alzheimer’s disease classification, demonstrating the advantages of combining preprocessing and transfer learning. Khan et al. (2022) used gray matter extraction from MR images of the ADNI database and a pretrained VGG network. By adding more network layers and using a layerwise freezing strategy for convolutional blocks, a transfer learning approach was implemented, which achieved strong classification performance in small sample scenarios. Mehmood et al. (2021) implemented a layerwise transfer learning strategy using the VGG architecture for multiclass classification among normal control, MCI, and Alzheimer’s disease. Interestingly, the model achieved an accuracy of 0.987 for Alzheimer’s disease *versus* normal control discrimination (Mehmood et al., 2021).

However, these methods have weak discriminative ability. To improve classification accuracy, researchers have attempted to address this problem by incorporating multimodal data, fine-tuning network structures, or adversarial calibration methods. For example, Wang et al. (2022b) applied a stacked autoencoder to integrate structural MRI features with frontal-parietal metabolite information, and the trained model obtained an accuracy of 0.98–1.00, an AUC of 0.99–1.00, and a specificity of 0.95–1.00 (Wang et al., 2022b). Park et al. (2023) designed an interpretable deep model, TabNet, with an AUC of 0.951 in the Alzheimer’s disease versus normal control classification task. To alleviate the intersite variability in multicenter MRI datasets, Cobbinah et al. proposed a two-stage deep learning framework. Specifically, a convolutional adversarial autoencoder is applied in the first stage to align images across sites, and then a convolutional residual attention network is trained to classify Alzheimer’s disease, MCI, and normal control. The model achieved classification accuracies of 0.918, 0.901, and 0.881 across three binary classification tasks, consistently outperforming traditional preprocessing pipelines (Cobbinah et al., 2022). In summary, these multimodal fusion and cross-domain calibration strategies can not only improve the accuracy of classification models but also enhance their generalizability in heterogeneous data environments.

Building on this research, a growing number of recent studies have demonstrated that applying structural segmentation as a preprocessing step can lead to more accurate classification results. For example, on the basis of their evaluation of commonly used structural segmentation tools such as SynthSeg and TigerBx, Wang et al. (2024) showed that when applied to high-quality segmentation output, their discriminative model achieved significantly better performance on the downstream classification task. This work highlights the importance of structural segmentation as a preprocessing step in image-based diagnostic workflows. After lesion segmentation, Jin et al. (2024) proposed a modified MobileNetV3-based classification model with attention mechanisms, dilated convolutions, and transfer learning to enhance lesion-aware representation. The classification accuracies of the proposed model on five tasks were 0.926–0.979 higher than those of the original architecture, which ranged from 2.6%–3.1%. Rao et al. (2025) proposed a two-stage diagnostic system constructed upon segmentation-based preprocessing. The system uses a multiscale attention Siamese network as a preprocessing stage to extract both local and global features from the input images. It utilizes the slime mold algorithm to optimize feature selection and applies EfficientNetB7 to classify the stages of Alzheimer’s disease. The model achieved classification accuracies of 0.993 and 0.994 on the Kaggle and ADNI datasets, respectively.

Subsequent studies have integrated structural segmentation with feature extraction, feature selection, and classification into unified multistage frameworks to enable end-to-end optimization of Alzheimer’s disease diagnosis. For example, Tuan et al. (2022) utilized brain tissue regions obtained from segmentation as input to a hybrid classification model combining XGBoost and SVM. The model achieved accuracies of 0.88 and 0.80 on the AD-86 and AD-126 datasets, respectively. Gowsikraja et al. (2025) proposed a novel framework, SBERO_Deep SNN, which integrates image segmentation via UNeXT with the SBERO optimization algorithm and leverages a deep spiking neural network for Alzheimer’s disease classification. The model achieved an accuracy of 0.905, sensitivity of 0.900, and specificity of 0.902, outperforming several mainstream techniques in early-stage diagnosis (Gowsikraja et al., 2025). Nithya et al. (2024) used K-means clustering for brain tissue segmentation and utilized ResNet-50 to extract discriminative features for classification. The model achieved an accuracy of 0.95 in Alzheimer’s disease recognition and effectively mitigated overfitting (Nithya et al., 2024). Maity et al. (2024) evaluated multiple classifiers using functional features for Alzheimer’s disease status classification. Among the tested combinations, VGG-16 paired with a random forest (VGG-16-RF) yielded the best performance, achieving an accuracy of 0.969 (Maity et al., 2024). Vinukonda and Jagadesh (2025) used an enhanced DeepLabV3^+^ for lesion segmentation, followed by feature extraction via LeNet-5 and stagewise classification of Alzheimer’s disease via an augmented ResNeXt model. The model achieved an overall accuracy of 0.981 and an AUC of 0.97 for moderate dementia classification, demonstrating superior performance compared with existing approaches (Vinukonda and Jagadesh, 2025).

In summary, image classification plays a pivotal role in the automated diagnosis and clinical staging of Alzheimer’s disease. CNN-based and extended classification models have made significant advancements in recognition accuracy and feature representation. Transfer learning and multimodal fusion strategies have further improved model robustness. Additionally, segmentation-assisted classification frameworks have considerably enhanced performance. However, existing methods still encounter challenges in few-shot learning, early lesion detection, and model interpretability. Future research could focus on longitudinal temporal modeling and disease trajectory prediction by integrating follow-up imaging data with cognitive assessments to better characterize the progression of Alzheimer’s disease.

Based on the findings above, the diffuse cortical changes observed in Alzheimer’s disease are particularly well captured by global context-oriented architectures such as 3D CNNs or Vision Transformer models, and their performance can be further improved through the multimodal fusion of MRI and positron emission tomography (PET) data. Emerging paradigms such as self-supervised pretraining, multimodal fusion of MRI and PET, and graph-based approaches for modeling network-level pathology, along with explainable AI frameworks, are expected to significantly enhance early detection, prognostic prediction, and clinical translation in Alzheimer’s disease. While deep learning has shown considerable potential in MRI-based lesion segmentation and image classification for Alzheimer’s disease, this condition is not the only neurodegenerative disorder associated with cognitive impairment. To further assess the generalizability and adaptability of deep learning methods in this field, it is crucial to extend the analysis to other causes of cognitive impairment. Parkinson’s disease, another neurodegenerative disorder characterized by structural brain alterations and cognitive deficits, serves as an additional representative case. The following section reviews the application of deep learning techniques to medical image analysis for Parkinson’s disease.

## Deep Learning Methods for Brain Magnetic Resonance Imaging Analysis in Parkinson’s Disease

Building on the preceding discussion of deep learning applications in Alzheimer’s disease, another major neurodegenerative disorder that warrants attention is Parkinson’s disease. Parkinson’s disease is a common neurodegenerative disorder characterized primarily by motor symptoms such as resting tremor, muscular rigidity, and bradykinesia (Wiesman et al., 2025). In the middle to late stages of the disease, patients frequently experience a range of nonmotor symptoms, including cognitive impairments such as executive dysfunction, reduced attention, and diminished verbal fluency (Gerner et al., 2025). A neuroimaging study has revealed that patients with Parkinson’s disease often exhibit varying degrees of structural atrophy or functional abnormalities in brain regions such as the substantia nigra and basal ganglia, suggesting that Parkinson’s disease-related cognitive impairment has a well-defined neuroanatomical basis (Hjelle et al., 2025). In recent years, deep learning has been increasingly applied to Parkinson’s disease-related medical image analysis owing to its powerful ability to automatically extract high-dimensional and complex features. Owing to the lower dependence of classification tasks on precise lesion localization, they play a key role in the early diagnosis and disease staging of Parkinson’s disease. In contrast, research on lesion segmentation and object detection is relatively limited, as Parkinson’s imaging typically lacks clearly identifiable structural lesions. Therefore, this section focuses on recent advances in image classification for Parkinson’s disease imaging analysis.

### Lesion segmentation and object detection in Parkinson’s disease magnetic resonance images via deep learning

In recent years, deep learning techniques have been increasingly applied to the analysis of imaging in Parkinson’s disease. Researchers have explored image segmentation methods to accurately identify key brain regions, such as the substantia nigra and globus pallidus, and have utilized object detection techniques to automatically localize potential lesion areas, aiming to provide effective support for clinical diagnosis and disease assessment. As shown in **Additional Table 12**, Dünnwald et al. (2021) developed an automated segmentation pipeline based on a 3D U-Net architecture, achieving a mean localization error of 2.2 mm in patients with Parkinson’s disease, which demonstrates strong performance. Gaurav et al. (2022) proposed NigraNet, an automated segmentation framework built on a modified U-Net architecture for delineating the substantia nigra pars compacta in MR images, achieving a Dice coefficient of 0.80 and an AUC of 0.85. Solomon et al. (2021) introduced the GP-net model, which can achieve high-precision 3D segmentation of the globus pallidus on 7T MRI, outperforming atlas-based methods in both quantitative and qualitative tasks. For object detection, Chen et al. (2024) combined YOLO-v5, LeNet, and radiomic features to create a hybrid model, demonstrating that their approach can achieve an automatic classification accuracy greater than 0.95 for early Parkinson’s disease. Li et al. (2024) enhanced the YOLOv5 architecture with an attention mechanism and dynamic convolution, resulting in superior performance compared to baseline methods in terms of accuracy, mAP, and recall. Despite the promising results of these methods, their application in Parkinson’s disease remains experimental due to challenges such as ambiguous anatomy, difficulties in annotation, and limited generalizability. Therefore, as discussed in the following subsection, recent advances in image classification for Parkinson’s disease imaging analysis will be introduced.

**Additional Table 12:** Summary of deep learning models applied in Parkinson's disease

Studies	Models	Innovative aspects	Research results	Practical significance
Kiryu et al., 2019	CNNS	Convolutional neural networks	Accuracy: 0.968; AUC: 0.995	CNNs enable accurate and automated classification of PD, PSP, MSA, and HC.
Chakraborty et al., 2020	3D CNNs	3D convolutional neural network	Accuracy: 0.953; Precision: 0.927	3D CNNs based on T1WI enable accurate early classification and detection of PD.
Dünnwald et al., 2021	3D-Unet	Several 3D-Unet-based CNNs	DS: 2.2 mm	A 3D U-Net-based automated segmentation pipeline provides accurate localization for PD patients.
Solomon et al., 2021	GP-net	Automatic segmentation	DS: 0.83	GP-net enables precise 3D segmentation of the globus pallidus on 7T MRI, outperforming atlas-based methods.
Yasaka et al., 2021	CNNs	Parameter-weighted connectome matrices	AUC: 0.895	CNNs with DKI-based structural connectivity matrices effectively distinguish PD patients from HCs.
Gaurav et al., 2022	NigraNet	A fully automatic SNc segmentation	DS: 0.80; AUC: 0.85	NigraNet, a modified U-Net framework, enables accurate MRI-based segmentation of the substantia nigra pars compacta.
Safai et al., 2021	GAT	End-to-end graph attention network	Accuracy: 0.86; F1 score: 0.86	A graph attention network integrating multimodal MRI connectomic features enhances PD classification performance.
Pahuja and Prasad, 2022	CNN SSAE	Multimodal features	Accuracy: 0.933; Accuracy: 0.924	A CNN and stacked sparse autoencoder fusion framework enables early PD classification using multimodal data.
Erdaş Ç and Sümer, 2023	2D CNNs 3D CNNs	Downsampled whole-brain volume	Accuracy: 0.962; Precision: 0.941	Integrating 2D and 3D CNNs enhances automated PD classification from T1WI images.
Wang et al., 2023b	CNNs SE- ResNeXt50	Multiple attention mechanisms	DS: 0.83; AUC: 0.901	The CNN-SE-ResNeXt50 framework offers an effective tool for accurate PD diagnosis.
Song et al., 2023	CNNs ViT	Segmentation and classification	DS > 0.85 AUC > 0.8	The CNN-Vision Transformer model enables accurate and efficient PD diagnosis.
Chen et al., 2024	YOLO-v5 LeNet	A novel hybrid model	ACC (external): 0.958	The YOLO-v5 and LeNet hybrid model enables accurate early PD classification.
Li et al., 2024a	YOLOv5	Predicting and classifying	Precision: 0.961; mAP: 0.986	The attention and dynamic convolution-enhanced YOLOv5 improves PD detection accuracy.
Welton et al., 2024	Heuron IPD Heuron NI	Quantitative susceptibility	AUC (IPD): 0.92; AUC (NI): 0.90	N1-guided deep learning with 3T MRI improves PD diagnosis.
Majhi et al., 2024	VGG16 DenseNet InceptionV3	GWO automatically fine-tunes the hyperparameters	Accuracy: 0.999; AUC: 0.999	Hyperparameter tuning with the grey wolf optimizer enhances hybrid CNN models for highly accurate PD classification.
Priyadharshini et al., 2024	3D CNNs	Whale optimization	Accuracy: 0.97	Deeply nested 3D-CNNs with feature fusion and whale optimization improve PD classification performance.
Patil and Ford, 2024	DcCNN FE-DcCNN	A multiscanner dataset	Accuracy: 0.778	The FE-DcCNN model addresses class imbalance with decorrelation optimization, enabling unbiased automatic PD classification.
Acikgoz et al., 2024	SE-ResNeXt	Densely connected and residual learning	Accuracy: 0.944; Precision: 0.917	SE-ResNeXt improves Parkinson's disease classification using dense connections, residual learning, and attention.
Chang et al., 2025	ResNet18	Different modal images	Accuracy: 0.97; Precision: 0.93	A ResNet18-based PET/MRI fusion model enhances the classification of PD, MSA, and HC.
Alrawis et al., 2025	FCN-PD	Capturing of local spatial details	Accuracy: 0.972; Accuracy: 0.956	CN-PD with EfficientNet and attention enhances PD classification.
Goyal et al., 2025	Five DL models	Deep learning-based ensemble technique	Accuracy: 0.979	The ensemble method with weighted ranking improves multi-class PD classification.
Kumar et al., 2025	Neuro_DeFused-Net	Multi-scale 2D CNNs and deep feature-level fusion	Accuracy: 0.971; AUC: 0.99; mAP: 0.995	Multi-scale 2D CNNs with feature fusion enhance PD classification using structural and functional MRI.

This table summarizes representative deep learning methods for PD, covering classification, segmentation, and detection tasks. The models utilize diverse architectures such as CNNs, U-Net variants, graph-based networks, and attention mechanisms to improve diagnostic accuracy, enable automated lesion detection, and support early disease identification and monitoring in clinical settings. AUC: Area under the curve; CNNs: convolutional neural networks; DS: dice score; FCN-PD: fully convolutional network for Parkinson's disease; GAT: graph attention network; GP-net: globus pallidus network; GWO: grey wolf optimizer; HC/HCs: healthy control(s); IPD: idiopathic Parkinson's disease; mAP: mean Average Precision; MRI: magnetic resonance imaging; MSA: Multiple system atrophy; NI: normal imaging; PD: Parkinson's disease; PSP: progressive supranuclear palsy; SE-ResNeXt: squeeze-and- excitation ResNeXt; SSAE: stacked sparse autoencoder; T1WI: Tl-weighted imaging; T2WI: T2-weighted imaging; VGG: visual geometry group; YOLO: you only look once.

### Image classification in Parkinson’s disease magnetic resonance images with deep learning

Due to the advantages mentioned above, image classification has increasingly demonstrated its significance in Parkinson’s disease imaging research. Researchers have trained deep neural networks, primarily CNNs, to automatically learn and detect latent pathological patterns from images, allowing them to classify Parkinson’s disease patients and healthy controls or to distinguish Parkinson’s disease patients from those with other Parkinsonian syndromes. Consequently, the time-consuming and variable classification process across subjects can be completed automatically and accurately by CNNs. As shown in **Additional Table 12**, Yasaka et al. (2021) constructed a parameter-weighted structural connectivity matrix from diffusion MRI and then trained CNNs to differentiate Parkinson’s disease patients from healthy controls. Among the three weighting methods evaluated, the matrix based on diffusion kurtosis imaging exhibited the best performance, achieving an AUC of 0.895. Kiryu et al. (2019) applied CNNs to automatically classify Parkinson’s disease, progressive supranuclear palsy, multiple system atrophy with predominant parkinsonism, and healthy controls, achieving an accuracy of 0.968 and an AUC of 0.995, demonstrating strong discriminatory performance. To fully preserve the spatial structure of MRI data, Chakraborty et al. (2020) utilized 3D CNNs based on T1-weighted images (T1WI) for the early automatic classification and detection of Parkinson’s disease, achieving a classification accuracy of 0.953 and a mean precision of 0.927. Subsequently, Erdaş and Sümer (2023) integrated both 2D and 3D CNNs to train on preprocessed T1WI images, attaining an accuracy of 0.962 and a precision of 0.941 in the automatic classification of Parkinson’s disease, thereby validating the effectiveness of multidimensional CNNs in this context. To facilitate collaborative decision-making across multiple representational hierarchies, Ke et al. (2024) employed multidimensional CNNs to extract tensor-based features and integrated decision outputs through a multibranch CNN architecture, enabling high-precision classification of Parkinson’s disease.

To further enhance the capacity of models for key feature extraction and classification performance, researchers have continuously refined and optimized CNN architectures. For instance, Pahuja and Prasad (2022) developed a deep learning model that integrates CNNs with stacked sparse autoencoders to facilitate the early classification of Parkinson’s disease using T1-weighted imaging (T1WI), SPECT, and cerebrospinal fluid data. Among the various methods evaluated, the CNN-based feature-level fusion framework achieved the highest accuracy of 0.933. Following this, Priyadharshini et al. (2024) introduced a deeply nested 3D-CNN that incorporates feature fusion and whale optimization algorithms, which boosted the classification accuracy of Parkinson’s disease to 0.97. To tackle the issue of class imbalance, Patil and Ford (2024) developed a decorrelated convolutional neural network (DcCNN). This model incorporates a decorrelation-based optimization function and a feature extraction module to create the FE-DcCNN model, enabling automatic classification of Parkinson’s disease while minimizing bias. To obtain a more comprehensive representation of multidimensional imaging features, Kumar et al. (2025) employed multiscale 2D CNNs and conducted deep feature-level fusion of structural and functional MRI data. Their model achieved a classification accuracy of 0.971, an F1 score of 0.976, and an AUC of 0.99. Additionally, to improve the extraction of key brain region features, Alrawis et al. (2025) developed the FCN-PD model, which combines EfficientNet with attention mechanisms. The model demonstrated classification accuracies of 0.972, 0.956, and 0.968 on the PPMI, OASIS, and MIRIAD datasets, respectively, highlighting its overall superiority over conventional models.

The abnormal regions in imaging related to Parkinson’s disease are often small and scattered across various spatial locations. Therefore, conducting structural segmentation before classification can help highlight important brain regions while suppressing irrelevant background information. This approach can significantly enhance the accuracy and robustness of subsequent classification efforts. Following this rationale, Wang et al. (2023b) were the first to utilize CNNs for the automatic segmentation of deep gray matter structures in the brain. They then constructed an SE-ResNeXt50 model with an attention mechanism to classify Parkinson’s disease patients and healthy subjects. The segmentation stage achieved an average Dice coefficient greater than 0.83 across all brain structures, and the final classification AUC reached 0.901, demonstrating the model’s effectiveness in both segmentation and classification tasks. Similarly, Song et al. (2023) combined CNNs with a vision transformer to automatically segment brain structures and classify Parkinson’s disease patients and healthy subjects. Their models achieved Dice coefficients exceeding 0.85 across all brain structures and AUC values greater than 0.8 during the classification stage. Additionally, they noted a significant reduction in segmentation time compared to conventional methods, underscoring the model’s strengths in both accuracy and computational efficiency.

In addition to classical CNNs and their improved variants, various other deep learning approaches have been applied to the automated analysis of brain MR images in Parkinson’s disease. For instance, Safai et al. (2021) proposed a graph attention network model that integrates multimodal connectomic features from structural and functional MR images for Parkinson’s disease classification. Using 10-fold cross-validation, this model achieved an accuracy and F1 score of 0.86, indicating strong classification performance. Additionally, Welton et al. (2024) evaluated two nigrosome-1 (N1)-guided deep learning models using 3T MR images. The Heuron IPD model focused on morphological abnormalities in the N1 region and achieved an AUC of 0.92, while the Heuron NI model utilized volumetric features of N1 and achieved an AUC of 0.90. In the realm of multimodal learning, Chang et al. (2025) introduced a ResNet18-based model that use.s PET/MRI imaging to classify Parkinson’s disease patients, patients with multiple system atrophy, and healthy controls. In cross-validation, the model that fused CFT-PET and ADC-MRI data demonstrated the best performance, achieving an accuracy of 0.97 and an AUC of 0.96. To enhance feature extraction capabilities, Acikgoz et al. (2024) developed an SE-ResNeXt network that incorporates dense connections, residual learning, and attention mechanisms. This model achieved an accuracy of 0.944 on the T2-weighted imaging (T2WI) dataset. Notably, some studies have introduced hybrid models to further improve classification performance. For example, Majhi et al. (2024) applied the gray wolf optimizer for hyperparameter tuning on VGG16, DenseNet, InceptionV3, and their combinations. Among these, the hybrid model GWO-VGG16 + InceptionV3 achieved the highest performance, with an accuracy of 0.999 and an AUC of 0.999. Goyal et al. (2025) proposed a deep learning ensemble method based on a weighted ranking strategy, which included a three-stage pipeline consisting of preprocessing, model training, and fusion-based decision-making. In the multiclass classification task for Parkinson’s disease, this model achieved an accuracy of 0.979.

In summary, the application of deep learning in Parkinson’s disease imaging analysis has made notable progress, with classification-oriented backbones such as ResNet and Vision transformer (ViT) and region-guided attention mechanisms being particularly effective in capturing the subtle and spatially distributed abnormality characteristics of the disease. CNN-based classification approaches and their variants have advanced structural modeling, feature representation, and multiclass discrimination, whereas segmentation-assisted classification strategies have helped reduce feature redundancy and improve robustness. Despite these advances, current research still faces significant challenges, including limited sample sizes, a strong reliance on labeled data, insufficient interpretability, and constraints on clinical deployment (Martinez-Murcia et al., 2017). In Parkinson’s disease, graph-based deep learning for connectome-level analysis, fine-grained modeling of small and spatially distributed abnormalities, and explainable AI frameworks are emerging as key paradigms to increase diagnostic sensitivity and ensure reliable deployment in clinical settings. Future work should focus on multimodal information fusion and structure–function coupling models to better characterize deep and small brain structures, thereby enhancing diagnostic accuracy and facilitating clinical translation.

## Current Status of Clinical Translation and Prospects for Practical Applications

In recent years, AI, particularly deep learning technology, has increasingly found applications in neuroimaging, transitioning from theoretical exploration to clinical translation. This trend shows notable promise in addressing neurological disorders associated with cognitive impairment, including ischemic stroke, Alzheimer’s disease, and Parkinson’s disease. As of 2024, several representative trials have been registered on ClinicalTrials.gov, including NCT071138495, NCT05959746, and NCT06295263, which focus on the diagnosis of stroke, dementia, and geriatric diseases. These studies encompass a spectrum of research, ranging from early-phase exploratory investigations to multicenter randomized controlled trials. As highlighted in **Box 1**, this trend underscores the growing clinical significance of AI in diagnostic support, disease prediction, and personalized medicine (Wang et al., 2022a). Simultaneously, the U.S. Food and Drug Administration (FDA) has been expediting the regulatory evaluation of AI-driven medical technologies. By August 2024, the FDA had approved nearly 950 devices that incorporate AI or machine learning, with approximately 77% deployed in radiology and about 3% in neurology (Joshi et al., 2024). For instance, an AI-assisted platform developed by Viz.ai, Inc. enables real-time analysis of brain CT scans, autonomously detects large vessel occlusions, and promptly alerts specialists remotely. This system, which has received FDA approval, has been implemented in clinical settings to improve response times and enhance the efficiency of stroke interventions. In the realm of research funding, the U.S. National Institutes of Health (NIH) has actively supported the clinical translation of AI-integrated neuroimaging research. Notable initiatives include a deep neural network-based MRI reconstruction framework developed by The Ohio State University, as well as a $2.3 million grant awarded to Indiana University for the creation of an open-source AI platform for brain imaging analysis. Furthermore, several international neuroimaging databases have provided critical resources for training and validating AI models. For example, the Alzheimer’s Disease Neuroimaging Initiative (ADNI), launched in 2004, has systematically collected data related to Alzheimer’s disease, including MRI, PET, biomarkers, and cognitive assessments. The Human Connectome Project (HCP) offers large-scale, high-resolution structural and functional brain connectivity data, facilitating the identification of brain network structures through deep learning models. The UK Biobank integrates multimodal MRI, genetic, and behavioral data, enabling the development of cognitive disorder diagnostic models with high generalizability.

**Box 1:** Current status of clinical translation of artifical intelligence (AI) in neuroimaging for cognitive disorders

**• ClinicalTrials.gov registered projects:**
- NCT07138495: Integrating AI in stroke neurorehabilitation (not yet recruiting). - NCT05959746: Clinical development of an AI tool for stroke diagnosis (recruiting). - NCT06295263: Application of AI technology for the diagnosis and treatment of geriatric diseases (recruiting).
**• NIH and international funding projects:**
- NIH: National Institutes of Health, USA (grant No. 1RF1MH126732) – Indiana University, $2.3M project for development of an open-source AI brain imaging analysis platform. - EU: Horizon 2020 AI-MIND project (grant No. 964220) – Early dementia risk prediction using AI-driven tools, coordinated by Oslo University Hospital. - China: National Key R&D Program of China (Grant No. 2024YFC3507100) – Artificial intelligence-assisted brain science and cognitive disorder diagnosis.
**• Classic clinical trials & Safety:**
- ALERT Trial (NCT04142879, multicenter RCT): Viz.ai, Inc. large vessel occlusion (VO) triage software significantly reduced treatment times for stroke patients, with no device-related safety issues reported. - SYNCHRONISE Study (NCT04608617, multicenter): AI-assisted stroke triage improved door-to-puncture and workflow efficiency, with safety comparable to standard care. - AI-assisted amyloid PET in MCI/AD (NCT05383053): AI tool predicted amyloid positivity, reduced unnecessary PET scans, and showed improved diagnostic accuracy without added risk.
**• FDA approvals of AI tools:**
- As of 2025, nearly 950 AI-enabled medical devices have received FDA clearance, with approximately 77% focused on radiology and a small proportion on neurology applications. - Viz.ai LVO Detection (2018): AI-based LVO detection on CT; enables alerts to expedite stroke intervention. - Aidoc ICH Detection (2018): AI algorithm flags intracranial hemorrhage on CT, improving emergent triage efficiency.

The clinical translation of AI in neuroimaging analysis for cognitive disorders has demonstrated a positive shift from “methodological research” to “clinical deployment.” However, large-scale implementation still encounters several challenges, including model reliability, privacy protection, cross-center generalizability, and medical ethics (Köchert et al., 2024). Future research should focus on enhancing multicenter validation and regulatory harmonization to facilitate the practical adoption and standardization of AI tools in the diagnosis and treatment of cognitive disorders.

## Limitations

There are still several limitations in the current research on MRI-based deep learning approaches for investigating cognitive impairments, including the following: (1) Lack of interpretability of the results. Traditional statistical methods can provide an explicit explanation for every step of input data processing. However, deep learning usually uses nonlinear processing, and the data analysis procedure is similar to a ‘’black box’’ (Bhagawati et al., 2023). Although deep learning can achieve higher computational accuracy because of the large networks and millions of parameters, the large structure also hinders the interpretability of the input-output relationship. (2) High dependence on data volume. Deep learning methods usually require a large amount of labeled data for training. If the training data are insufficient, overfitting is likely to occur, and the generalization ability of the model will be affected. (3) Poor ability to detect small lesions. Although the deep learning method has achieved outstanding performance in large-scale lesion detection, it still faces great challenges in detecting small lesions. Especially in medical images, high-resolution and high-accuracy requirements pose great challenges for detecting small lesions. (4) Clinical validity should be verified. Currently, the evaluation criteria for deep learning focus on theoretical accuracy. However, the final evaluation should be based on its clinical validity, that is, the value and effect of deep learning on clinical applications and decision-making. (5) Medical data quality and availability issues. The quality of medical data is often low, and some data even have missing values or inaccurate annotations. In addition, medical image data contain a large amount of sensitive information, and their sharing involves privacy protection and legal issues, which affect the availability of data (Fernandes et al., 2024a). The above issues may introduce biases in the training process, which will affect the model’s training outcome and practical application.

## Future Research Directions

To overcome the above limitations, future research directions can be summarized as follows: (1) Increasing model interpretability: To make black-box models more interpretable, future research can focus on more interpretable algorithm designs. For example, people can design attention-based algorithms, visualization methods, or interpretable models to explore deep model decision-making processes in certain tasks (Fernandes et al., 2024b). In addition, by combining rule learning and clinical expert knowledge, deep learning models can not only achieve high accuracy but also provide solid explanations for their outputs. (2) Few-shot learning and innovative algorithms: Due to the limited amount of training data, deep learning models may lead to overfitting. Therefore, transfer learning methods can be utilized in future research to learn effective features from data. Furthermore, with the rapid development of computer hardware and innovative algorithms, the amount of training data can be augmented via synthetic and simulated data methods to improve the generalization performance of models, even when the data are limited. (3) Enhancing the detection of small lesions: High-resolution medical images, fine-grained analysis, and multiscale CNNs can be used to improve the accuracy of models in detecting small lesions. In addition, reinforcement learning and self-supervised learning methods can be used to enhance the fine-grained prediction ability of models in complicated scenarios. (4) Clinical validation and cross-domain applications: Our group will use deep learning algorithms to localize and find key regions related to cognitive impairments in ischemic stroke patients and validate the research through multicenter clinical trials and cross-domain datasets. The last step will be to modulate these regions to verify their clinical effectiveness. (5) Improving medical data quality and protecting data privacy: Future research should focus on improving the quality, accuracy, and consistency of medical data annotations to solve the problems of data quality and availability. For example, more efficient data annotation platforms can be developed, and semisupervised learning or active learning methods can be used to reduce the amount of manually annotated data while also improving data diversity and coverage. In addition, to solve the problem of data privacy, more research should be conducted on enhancing privacy protection technology and ensuring data privacy.

## Conclusion

This review synthesizes recent advances in deep learning-based brain imaging analysis for ischemic stroke, Alzheimer’s disease, and Parkinson’s disease from the perspective of cognitive-related disorders. Across these conditions, cognitive impairment patterns influence model design choices, from preprocessing strategies to network architecture selection. Ischemic stroke studies predominantly focus on segmentation tasks, using lesion-focused architectures such as U-Net or hybrid models to capture focal damage. Research on Alzheimer’s disease has emphasized classification and staging tasks, leveraging architectures with strong global context modeling, including transformers and 3D CNNs, to detect diffuse cortical atrophy. Parkinson’s disease imaging, which often lacks overt structural lesions, relies more on classification-oriented backbones such as ResNet or Vision transformers to detect subtle, distributed abnormalities. Despite notable progress, challenges remain in terms of data availability, interpretability, and clinical translation. Future research should focus on multimodal fusion, structure-function coupling, and cross-disease evaluations to identify transferable architectures and enhance model generalizability, ultimately advancing precision diagnosis and treatment of cognitive impairment.

## Additional files:

***Additional Table 1:*** *Inclusion and exclusion criteria.*

***Additional Table 2:*** *Comparison of emerging deep learning technologies for medical brain imaging analysis.*

***Additional Table 3:*** *Summary of CNN methods applied for lesion segmentation in ischemic stroke.*

***Additional Table 4:*** *Summary of U-Net methods applied for lesion segmentation in ischemic stroke.*

***Additional Table 5:*** *Summary of pure transformer methods applied for lesion segmentation in ischemic stroke.*

***Additional Table 6:*** *Summary of hybrid models applied for lesion segmentation in ischemic stroke.*

***Additional Table 7:*** *Summary of one-stage object detection methods used for object detection in ischemic stroke.*

***Additional Table 8:*** *Summary of two-stage object detection methods used for object detection in ischemic stroke.*

***Additional Table 9:*** *Summary of CNN methods applied for image classification in ischemic stroke.*

***Additional Table 10:*** *Summary of Transformer and hybrid models applied for image classification in ischemic stroke.*

***Additional Table 11:*** *Summary of deep learning models used in Alzheimer’s disease.*

***Additional Table 12:*** *Summary of deep learning models applied in Parkinson’s disease.*

## Data Availability

*All relevant data are within the paper and its Additional files.*
